# A Model for the Development of Alzheimer’s Disease

**DOI:** 10.1093/gpbjnl/qzaf087

**Published:** 2025-09-23

**Authors:** Zhenyu Huang, Xuechen Mu, Qiufen Chen, Lingli Zhong, Jun Xiao, Chunman Zuo, Ye Zhang, Bocheng Shi, Yingwei Qu, Renbo Tan, Long Xu, Renchu Guan, Ying Xu

**Affiliations:** College of Computer Science and Technology, Jilin University, Changchun 130012, China; Systems Biology Lab for Metabolic Reprogramming, Department of Human Genetics and Cell Biology, School of Medicine, Southern University of Science and Technology, Shenzhen 518055, China; Systems Biology Lab for Metabolic Reprogramming, Department of Human Genetics and Cell Biology, School of Medicine, Southern University of Science and Technology, Shenzhen 518055, China; School of Mathematics, Jilin University, Changchun 130012, China; Systems Biology Lab for Metabolic Reprogramming, Department of Human Genetics and Cell Biology, School of Medicine, Southern University of Science and Technology, Shenzhen 518055, China; Systems Biology Lab for Metabolic Reprogramming, Department of Human Genetics and Cell Biology, School of Medicine, Southern University of Science and Technology, Shenzhen 518055, China; College of Computer Science and Technology, Jilin University, Changchun 130012, China; Systems Biology Lab for Metabolic Reprogramming, Department of Human Genetics and Cell Biology, School of Medicine, Southern University of Science and Technology, Shenzhen 518055, China; Institute of Artificial Intelligence, Donghua University, Shanghai 201620, China; The First Laboratory of Cancer Institute, The First Hospital of China Medical University, Shenyang 110001, China; Systems Biology Lab for Metabolic Reprogramming, Department of Human Genetics and Cell Biology, School of Medicine, Southern University of Science and Technology, Shenzhen 518055, China; School of Artificial Intelligence, Jilin University, Changchun 130012, China; College of Computer Science and Technology, Jilin University, Changchun 130012, China; Systems Biology Lab for Metabolic Reprogramming, Department of Human Genetics and Cell Biology, School of Medicine, Southern University of Science and Technology, Shenzhen 518055, China; Northeast Asia Research Institute of Traditional Chinese Medicine, Changchun University of Chinese Medicine, Changchun 130012, China; Systems Biology Lab for Metabolic Reprogramming, Department of Human Genetics and Cell Biology, School of Medicine, Southern University of Science and Technology, Shenzhen 518055, China; College of Computer Science and Technology, Jilin University, Changchun 130012, China; Systems Biology Lab for Metabolic Reprogramming, Department of Human Genetics and Cell Biology, School of Medicine, Southern University of Science and Technology, Shenzhen 518055, China

**Keywords:** Alzheimer’s disease, Intracellular alkalosis, Extracellular acidosis, Metabolic reprogramming, Transcriptomic analysis

## Abstract

Intracellular alkalosis and extracellular acidosis are well-established characteristics of Alzheimer’s disease (AD). We present a computational analysis and modeling of transcriptomic data of AD tissues, aiming to understand their causes and consequences. Our analyses have revealed that (1) persistent mitochondrial alkalization is due to chronic inflammation coupled with elevated iron and copper metabolisms; (2) the affected cells activate multiple acid-producing metabolisms to keep the mitochondrial pH stable for survival; (3) the most significant one is the continuous import and hydrolysis of glutamine to glutamate, N$H_{3}$, and $H^{+}$, resulting in persistent release of glutamate, an excitatory neurotransmitter, into the extracellular space; (4) this leads to persistent hyperexcitability of the nearby neurons, resulting in their continuous firing and release of $H^{+}$-rich synaptic vesicles; (5) the $H^{+}$ is neutralized by bicarbonates released by the neighboring astrocytes in normal tissues, which could not keep up with the increased release of $H^{+}$ in their discharge rates of bicarbonates in AD tissues, leading to progressively increased extracellular acidosis and ultimately cell death; and (6) multiple extensively studied AD-associated phenotypes, including Aβ aggregates and tau fibers, are induced to help to alleviate the pH imbalances and beneficial to cell survival in the early phase of AD, which gradually become contributors to the AD development. Each step in this model is largely supported by published studies. Overall, we have developed a fundamentally novel and systems-level view of how AD may have evolved.

## Graphical abstract

**Figure:**
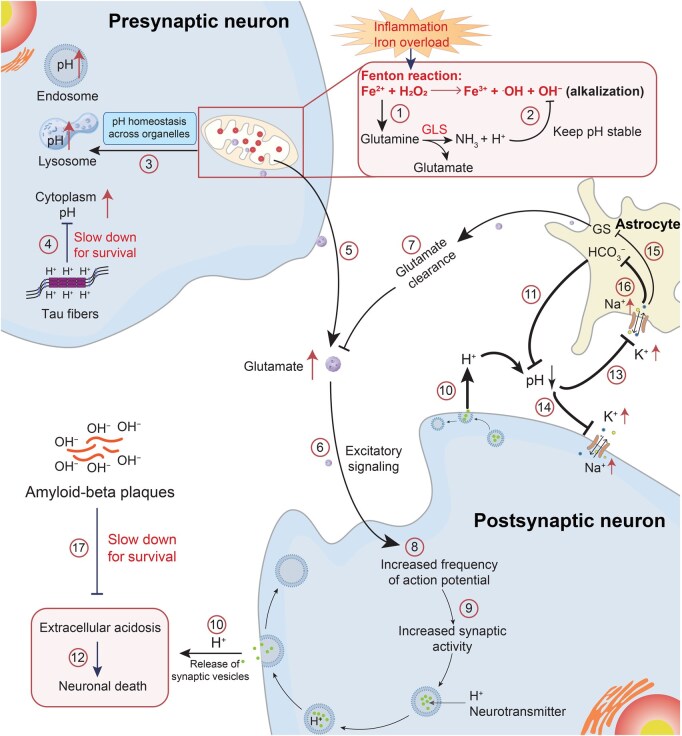

## Introduction

Alzheimer’s disease (AD) is a devastating and complex neurodegenerative disease. It starts with symptoms like dementia and gradually progresses to partial and then complete loss of key cognitive capabilities [1]. Currently, there is no treatment that can slow down the progression of the disease [2]. The most studied features of AD are deposits of amyloid-beta (Aβ) plaques and the formation of the neurofibrillary tangles (NFTs) by tau proteins [3,4]. Recent studies have revealed that AD has numerous fundamental changes in the chemical conditions as well as in cell biology, such as (1) elevated extracellular acidification and intracellular alkalization [5,6]; (2) altered homeostasis of key intracellular ions such as Na^+^ and $K^{+}$ [7,8]; (3) dysregulated metabolisms of zinc and copper [9]; (4) reduced glucose metabolism [10]; (5) progressively reduced amplitudes of synapses with increased frequencies [11]; (6) chronic inflammation and increased oxidative stress [12,13]; and (7) elevated neurotoxicity and extensive neuronal apoptosis [14,15].

While Aβ deposits and NFT formation have been the focus of extensive research in the past few decades, recent studies suggest they may not necessarily be the main reason for reduced cognitive capabilities [16,17]; instead, their formation relieves certain stresses initially [8,18]. The state-of-the-art understanding is that extensive neuronal death is the predominant reason for the reduced cognitive capacity in AD patients [19]. Although considerable amounts of information and data have been generated for AD, it represents a daunting task to sort out the causal relationships among the pathological changes and massive neuronal death.

Multiple proposals have been put forward about the possible causes for the extensive neuronal death in AD, including (1) oxidative stress that leads to damages to key molecules and the host neurons [12]; (2) extensive extracellular release of proinflammatory signals and neurotoxic free radicals [20]; (3) dysregulated intracellular pH that can repress functions of the acidic organelles like endosome and lysosome, resulting in detrimental effects to the host neurons [21,22]; (4) persistent extracellular acidification that represses the activities of the Na^+^/K^+^ exchanger, the sole exporter of the intracellular Na^+^, while the affected cells continue to import Na^+^, together leading to increased intracellular accumulation of Na^+^, which gives rise to reduced transmembrane potential as well as increased extracellular K^+^ level [23]; (5) synaptic losses associated with mis-localization of synaptic proteins, which can lead to reduced neuronal functions and cell death [19]; (6) the formation of tau aggregates can lead to cell death [24]; and (7) overexpression of amyloid precursor protein (APP) that can lead to Aβ-associated neuronal death in advanced AD tissues [24]. While each of these proposals can explain some of the behaviors of AD tissue cells, none provides a model for disease onset and progression that accounts for the majority of the AD phenotypes and is consistent with the available AD tissue omics data. Therefore, it is essential to establish a system-level model to understand the relationships among these factors and possibly others, leading to a detailed understanding of the main drivers and key molecular mechanisms of the disease.

Our study focuses on dysregulation of extracellular and intracellular pH homeostasis, as AD is known to cause persistent intracellular alkalization and extracellular acidification throughout the entire disease progression [25]. We aim to construct a model that explains the possible causes and consequences of pH imbalances and their roles in massive neuronal loss, which is consistent with the available transcriptomic data and established AD phenotypes. The first major challenge is the limited availability of transcriptomic data of AD tissues, particularly for mild cognitive impairment (MCI) tissues, namely pre-AD tissues. To address this issue, we assembled publicly available raw RNA-seq data using a novel transcript assembly pipeline, yielding considerably more high-quality transcripts than those available in the public domain, providing a solid foundation for our study.

We conducted analyses of these transcriptomic data and derived results that confirm some of the previous findings [18,23,26–28] and, more importantly, suggest the following: (1) neuronal cells in AD tissues harbor persistent Fenton reactions [18]:


(1) $${\mathrm{Fe}}^{2+}+H_{2}O_{2}\to {\mathrm{Fe}}^{3+}+\cdot \mathrm{OH}+{\mathrm{OH}}^{-}\;$$

in their mitochondria with superoxide（$\cdot O_{2}^{-}）$as the reducing molecule that converts ${\mathrm{Fe}}^{3+}$ back to ${\mathrm{Fe}}^{2+}$, hence driving continuous execution of the reaction and generation of OH^−^, where both $H_{2}O_{2}$ and $\cdot O_{2}^{-}$ are released by local astrocytes and diffuse inside the affected neurons; (2) in response to this persistent alkalization, multiple acidifying metabolisms are induced to produce H^+^, collectively at a rate comparable to the rate of OH^−^ production by mitochondrial Fenton reactions; (3) among them, persistent upregulation of glutaminase (GLS), widely observed in AD [29], hydrolyzes glutamine to glutamate and N$H_{4}^{+}$(= NH_3_ + H^+^), an acidic molecule; (4) while the H^+^ is used to neutralize the Fenton reaction-produced OH^−^, the glutamate is released extracellularly, which drives continuous firing of the neighboring neurons and releasing of H^+^-rich synaptic vesicles; (5) under normal conditions, the released H^+^ is neutralized by bicarbonates produced and released by the neighboring astrocytes [26]. In AD, however, the considerably increased rates of the neuron firing and H^+^ released, driven by the increased release of glutamates, could not be matched by the release of bicarbonates at the same rates by the astrocytes, hence resulting in progressively increased extracellular acidosis; (6) a major consequence of the persistent extracellular acidification is the repression of the Na^+^/K^+^ exchanger, leading to intracellular Na^+^ accumulation and increased extracellular K^+^ concentration, which further reduces the release rates of bicarbonates, hence together with (5) forming a vicious cycle and increased acidosis. As the acidosis persists, the affected neurons will die [28]; and (7) a very surprising observation is that the formation of NFTs helps to relieve the intracellular alkalosis. Similarly, the development of Aβ fibers slows down the extracellular acidification process in the early stage of AD.

**Figure 1:**
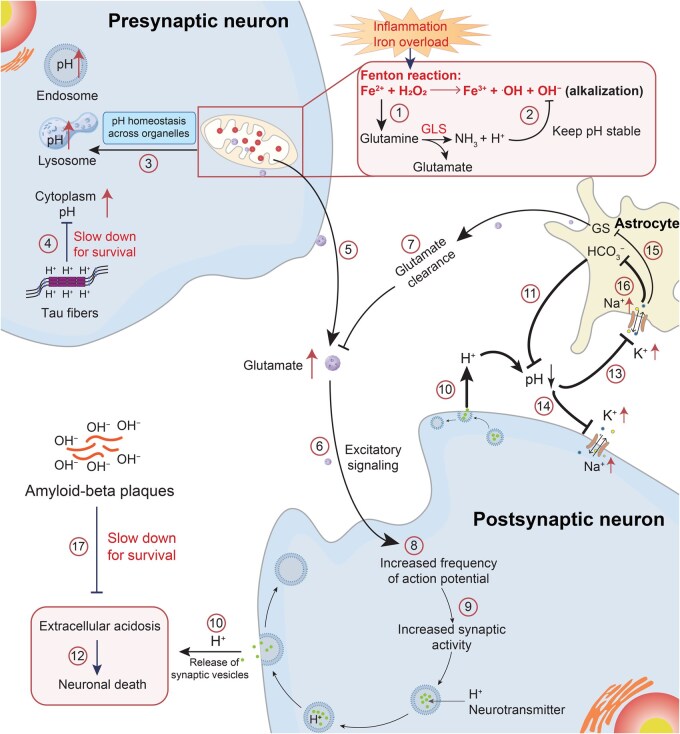
A schematic illustration of our model, consisting of key events and logical relationships **1**. Fenton reaction occurrence in mitochondria triggers elevated GLS expression; **2**. GLS-catalyzed reaction generates ${\mathrm{NH}}_{4}^{+}$ to neutralize intracellular alkalinity by Fenton reactions; **3**. Mitochondrial Fenton reaction-driven alkalinity leads to alkalization in cytosol, endosomes, and lysosomes; **4**. The tau protein hyperphosphorylation releases H^+^ to slow down cytoplasmic alkalization, as the symbol signifies; **5**. Glutamate, the main product of GLS-mediated reaction, is released into the extracellular space; **6**. Surrounding neurons uptake glutamates; **7**. Astrocytes clear glutamates released by Fenton reaction-affected neurons; **8**. Increased glutamate accumulation drives hyperactivation of nearby neurons; **9**. Activated neurons release synaptic vesicles; **10**. Released neurotransmitters, along with H^+^, enter the extracellular space, resulting in increased extracellular acidity; **11**. Astrocytes neutralize released H^+^ by releasing ${\mathrm{HCO}}_{3}^{-}$, which was slowed down due to increased concentration of extracellular K^+^, as a result of acidity-repressed Na^+^/K^+^ exchanger; **12**. Extracellular acidosis promotes cell death; **13**. and **14**. Extracellular acidity suppresses the Na^+^/K^+^ exchanger, leading to elevated levels of intracellular Na^+^ and extracellular K^+^ levels and consequently decreased levels of extracellular Na^+^ and intracellular K^+^ levels; **15**. A reduced Na^+^ level represses the rate of glutamate clearance, resulting in increased extracellular acidity; **16**. An increased extracellular concentration of K^+^, as result of repressed activity of Na^+^/K^+^ exchanger, leading to astrocyte depolarization and subsequent repression of ${\mathrm{HCO}}_{3}^{-}\;$export – note that steps 10, 11, 13, 15 form a vicious cycle leading to progressively increased extracellular acidification and ultimately massive neuronal death; **17**. Amyloid aggregates hide acidic amino acids, hence slowing the process of extracellular acidity, but their continuous formation disrupts cell-cell interaction, ultimately resulting in cell death. GLS, Glutaminase; GS, glutamine synthetase.

Based on these findings, we constructed a model to explain the onset and progression of AD. This model begins with inflammation-induced mitochondrial Fenton reactions and consists of multiple chemical imbalances and responding metabolic reprograms, ultimately leading to massive neuronal death (**Figure 1**). In this model, key relationships are first identified through statistical correlation analyses, and their causal links are inferred based on relevant chemical balance relationships, with further support by computational causal inference. The following sections provide detailed inference and analyses of these relationships.

## Results

### Fenton reactions in AD neuronal mitochondria

Previous studies have reported that AD tissue cells, or AD for simplicity, harbor Fenton reactions [30], but only in the context of discussing the high oxidative stress in AD, without further analyses of how Fenton reactions may have arisen and contributed to the disease development. Through transcriptomic data analyses of the MCI and AD tissues, we statistically demonstrated that persistent Fenton reactions occur in their mitochondria. This finding was established using a procedure we developed previously to show the occurrence of Fenton reactions in all cancer tissue cells [31]. We briefly outlined the idea of how we established that mitochondria of AD tissue cells have persistent Fenton reactions.

The Fenton reaction is an inorganic chemical reaction requiring no involvement of enzymes. It takes place when the concentrations of $Fe^{2+}$ and $H_{2}O_{2}$ reach beyond certain levels. The reaction persists when there are reducing molecules nearby that can convert $Fe^{3+}$ back to $Fe^{2+}$. We have previously demonstrated that cancer tissue cells generally use superoxide (${\cdot O}_{2}^{-})$ as the reducing molecule. When this happens, the reaction persists in the following form, also referred to as the Haber-Weiss reaction [31]:


(2) $${\cdot O}_{2}^{-}+H_{2}O_{2}\overset{Fe^{2+}}{\to }\cdot \mathrm{OH}+OH^{-}+O_{2}$$

with $Fe^{2+}$ serving as a catalyst and both ${\cdot O}_{2}^{-}$ and $H_{2}O_{2}$ being produced and released by macrophage and/or neutrophil, where $H_{2}O_{2}\;$enters the cells by diffusion via aquaporins [32] and ${\cdot O}_{2}^{-}$ via chloride channels [33], both of which can diffuse into mitochondria via the VDAC channels [34].

**Figure 2:**
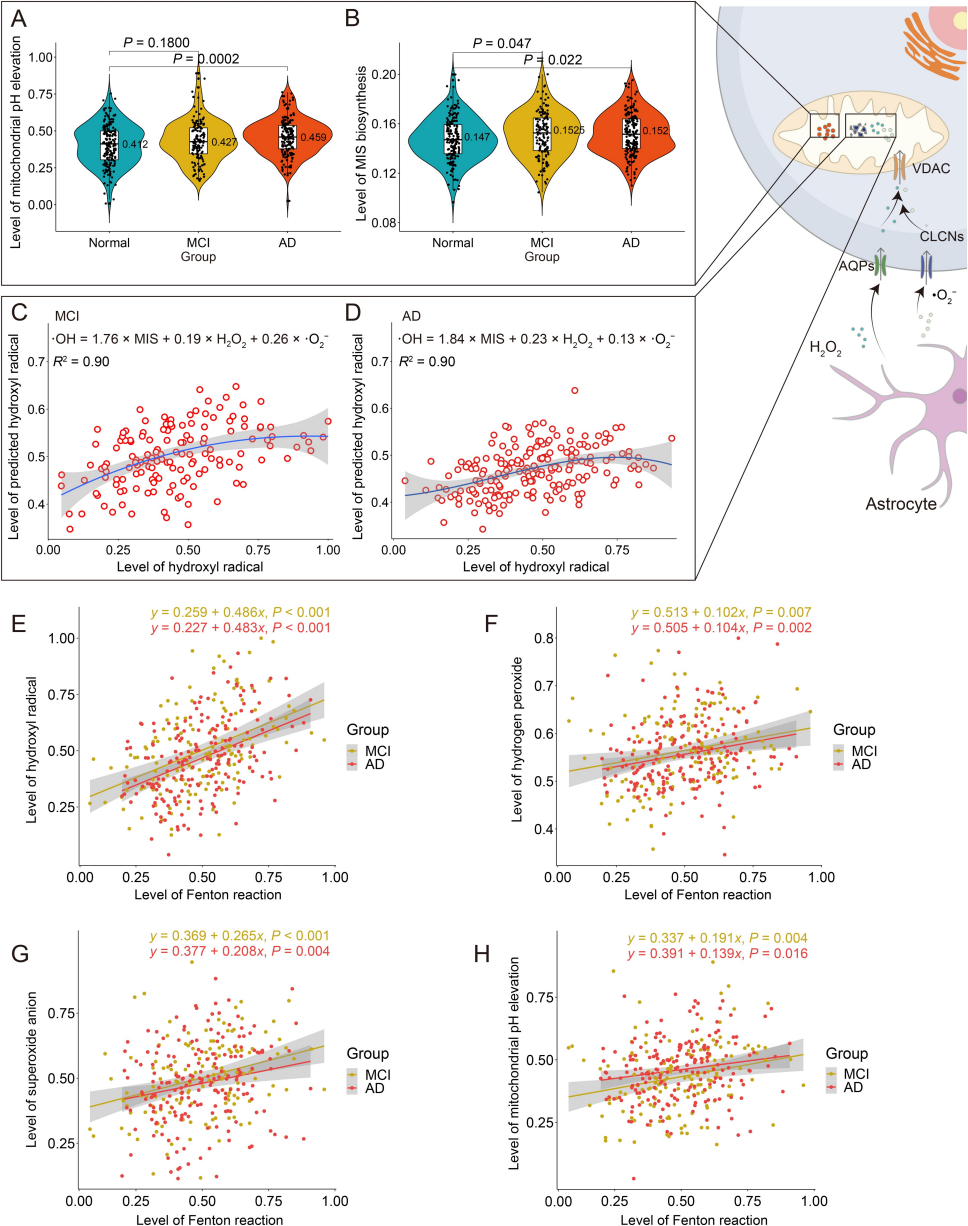
Evidence for the existence and significance of mitochondrial Fenton reaction **A**. Comparison between elevations in pH within mitochondria across three groups. The violin graph displays the means of mitochondrial pH for each group. **B**. Comparison of MIS cluster biosynthesis levels across three groups. Statistical significance was determined by Wilcoxon rank-sum test. **C**. Nonlinear regression analysis unveils that [·OH] can be represented as a function of the expression of $\left[H_{2}O_{2}\right]$, $\left[{\cdot O}_{2}^{-}\right]$, and MIS expression in the MCI group. **D**. Nonlinear regression analysis shows that [·OH] can be represented as a function of the expression of $\left[H_{2}O_{2}\right]$, $\left[{\cdot O}_{2}^{-}\right]$ and MIS expression in the AD group. **E**. Linear regression analysis shows a connection between ·OH production and level of mitochondrial Fenton reaction. **F**. Linear regression analysis reveals a relationship between level of mitochondrial Fenton reaction and hydrogen peroxide production. **G**. Linear regression analysis shows a connection between ${\cdot O}_{2}^{-}$ production and the level of mitochondrial Fenton reaction. **H**. Linear regression of the level of Fenton reaction and mitochondrial pH elevation. MIS, mitochondrial iron-sulfur cluster; VDAC, voltage-dependent anion channel; AQP, aquaporin; CLCN, chloride channel protein.

We discovered that mitochondria of AD tissue cells harbored (persistent) Fenton reactions using ${\cdot O}_{2}^{-}$ as the reducing molecule, in both MCI and AD tissues (**Figure 2**A), hence driving up the mitochondrial pH. For X = ·OH, $Fe^{2+},{\cdot O}_{2}^{-}$ or $H_{2}O_{2}$, its concentration [X] in mitochondria can be accurately represented as a nonlinear function of the expression of a small set of selected marker genes [35]. We have previously demonstrated that a subcellular compartment harbors persistent Fenton reaction “if and only if” [·OH] can be “accurately” represented as a function F() of [$Fe^{2+}],[{\cdot O}_{2}^{-}]$ and [$H_{2}O_{2}]$ based on the Michaelis-Menten equation of the above reaction (1) [31,35], where “accurately” is defined as that the two sides of the equation strongly and positively correlate having a statistical significance *P* < 0.05, using the expression of the marker genes whose proteins function in the same organelle. For simplicity, we defined $p^{M}$-value and $p^{A}$-value throughout the paper as the *P* values in MCI and AD samples, respectively.

We used the expression levels of the 26S proteosome genes to estimate [·OH], as it has been established that 26S proteosome is used specifically to degrade protein aggregates formed by reaction between ·OH and proteins [35], and ·OH can be produced only by Fenton reactions in the current context [31]. Hence, the expression levels of the 26S proteosome genes were used to reflect the level of Fenton reaction, which were considerably elevated in AD samples *vs*. in controls (Figure S1A). For the three other quantities, our analyses suggest that $Fe^{2+}\;$in iron-sulfur clusters is the iron responsible for mitochondrial Fenton reactions, where the iron sulfur-containing proteins are elevated in both MCI and AD samples *vs*. controls (Figure 2B), indicating that the iron-sulfur clusters damaged by ·OH need to be replaced. The genes for producing $H_{2}O_{2}$ and ${\cdot O}_{2}^{-}\;$by astrocytes were both significantly increased in AD *vs.* in controls (Figure S1B–E) [36].

By checking the aforementioned “if and only if” condition, we found that the estimated level of [·OH] strongly and positively correlated with $F(\left[{\mathrm{Fe}}^{2+}],[\cdot O_{2}^{-}],[H_{2}O_{2}\right])\;$with both $p^{M}$-value and $p^{A}$-value < 0.05, respectively. We thereby predict that mitochondria of the AD tissues under study habored persistent Fenton reactions (Figure 2C and D; Table S1).

To provide supporting evidence, we noted that (1) the expression of genes responsible for synthesizing iron-sulfur clusters strongly and positively correlated with those for 26S proteosome genes in both MCI and AD samples, which otherwise should be unrelated without Fenton reactions (Figure 2E); (2) the marker genes for $H_{2}O_{2}$ and ${\cdot O}_{2}^{-}\;$ were both strongly and positively correlated with the level of mitochondrial Fenton reactions with significant *P* values (Figure 2F and G); and (3) the expression of aquaporin genes (AQPs), chloride channel genes (CLCNs), and voltage-dependent anion channel (VDAC) genes each was strongly correlated with the level of Fenton reactions, with significant *P* values in both MCI and AD samples, as shown in **Table 1**, strongly suggesting that $H_{2}O_{2}$ and ${\cdot O}_{2}^{-}\;$diffuse through these channels into cytosol and then to mitochondria from the extracellular space where they are produced by astrocytes.

**Table 1:** Regression analysis of Fenton reaction levels against transporters/channels for H_2_O_2_ and ·$O_{2}^{-}$ transport

Quantity	MCI		AD	
	Coeff	*P* value	Coeff	*P* value
AQP2	0.828	5.66E−06	1.063	5.71E−12
AQP5	0.905	0.000114	0.228	0.0738
AQP6	0.448	0.00326	0.364	0.00154
CLCN2	0.429	0.00022	0.186	0.861
CLCN5	0.623	0.000894	0.506	0.00127
CLCNKA	0.462	0.00219	0.422	8.96E−05
VDAC	0.506	0.0706	0.794	1.80E−05
VDAC2	0.145	0.507	0.381	0.0198
VDAC3	0.429	0.0868	0.282	0.0980

*Note*: Quantity refers to channel proteins facilitating the entry of H_2_O_2_ and ·$O_{2}^{-}$ into cells. AQPs transport H_2_O_2_, while CLCNs transport ·$O_{2}^{-}$. Coeff represents the regression coefficient, and *P* value indicates the significance of the regression.

As expected, we observed that the increase in mitochondrial pH strongly correlated with the predicted level of mitochondrial Fenton reactions (Figure 2H). Furthermore, our analyses detected strong correlations between the level of mitochondrial Fenton reaction and the expression of both glutamine importer and exporter in MCI and AD samples (**Table 2**).

**Table 2:** Regression analysis of mitochondrial Fenton reaction and glutamine transporter expression

Group	Gene	Transcript	Regression F-statistic	DESeq2 *P* value	Regression Coeff	Log_2_ FC
MCI	*SLC1A2*	ENST00000643454.1	0.0117	0.049828	1.579653	0.267728
MCI	*SLC38A10*	ENST00000374759.8	0.00659	0.021221	1.840686	0.351788
MCI	*SLC38A10*	MSTRG.31529.6	0.0186	0.021115	1.588798	0.36398
MCI	*SLC25A1*	ENST00000659857.1	1.52E−03	0.02387	0.363938	0.552077
MCI	*SLC25A18*	MSTRG.45479.2	2.18E−02	0.001434	19.00402	0.326895
MCI	*SLC25A48*	MSTRG.57546.12	3.05E−02	0.024447	1.128609	0.34522
MCI	*SLC17A5*	MSTRG.60987.8	9.54E−05	0.818628	2.235706	0.0199
AD	*SLC7A2*	MSTRG.67306.7	0.00145	0.035657	4.555232	0.495199
AD	*SLC17A5*	MSTRG.60987.8	8.43E−03	0.026042	2.025324	0.173795
AD	*SLC38A7*	MSTRG.27034.17	0.04	0.043752	0.672264	0.187455
AD	*SLC6A12*	ENST00000540094.1	0.0321	1.17E−05	0.427537	0.424725
AD	*SLC6A1*	ENST00000645598.1	0.000505	6.85E−03	1.216845	0.328584
AD	*SLC6A12*	MSTRG.14072.11	0.0415	4.21E−03	3.973116	0.728098
AD	*SLC6A12*	MSTRG.14072.3	0.0234	3.49E−13	2.677424	0.811179
AD	*SLC25A48*	ENST00000412661.3	3.12E-02	0.000009	0.789301	0.568666

*Note*: Group refers to sample grouping. MCI represents mild cognitive impairment, and AD represents Alzheimer’s disease. Regression F-statistics is the *P* value of the F-test in linear regression. DESeq2 *P* value is the *P* value from the DESeq2 differential expression analysis. Regression Coeff denotes the linear regression coefficient, and Log_2_ FC represents the log_2_ fold change calculated by DESeq2.

All these data provided strong evidence supporting that (1) persistent Fenton reactions took place in mitochondria in both MCI and AD tissues, continuously producing OH^−^, and (2) the increased import of glutamine and its conversion to glutamate were strongly associated with and possibly driven by mitochondrial Fenton reactions (further evidence is provided in the next section). All relevant marker genes are listed in Table S2.

### Mitochondrial alkalization drives metabolic and cellular changes to keep pH stable

Our prediction that persistent Fenton reactions continuously generate net $OH^{-}$ is supported by the literature that AD cells have an elevated intracellular pH value, approximately 7.0 for MCI and 7.04 for AD, compared to the normal one at ∼ 6.8 [5]. Here, we evaluated how mitochondria maintain pH stability when harboring persistent Fenton reactions, which is essential for cell viability and survival. Specifically, we systematically examined all altered metabolisms that are pH relevant.

#### Acidifying metabolic reprogramming and importers

**Figure 3:**
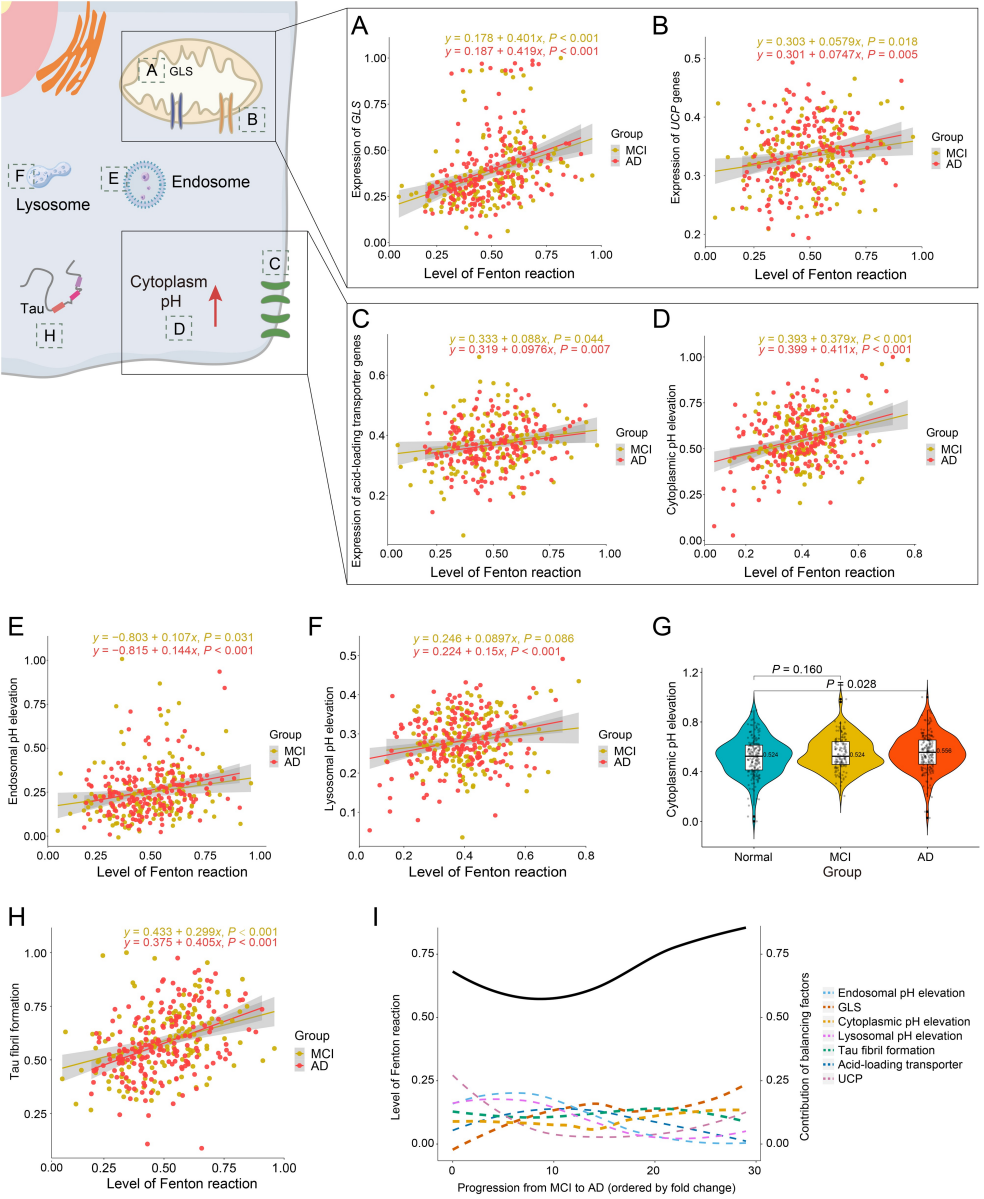
Reprogrammed metabolisms induced by mitochondrial Fenton reactions **A**. Linear regression analysis reveals a functional relationship between level of mitochondrial Fenton reaction (x-axis) and GLS expression (y-axis). **B**. Linear regression analysis shows correlation between the level of mitochondrial Fenton reaction (x-axis) and the combined expression of *UCP* genes (y-axis). **C**. Linear regression analysis depicts a functional relationship between the level of mitochondrial Fenton reaction (x-axis) and the expression of acid-loading transporter (y-axis) genes. **D**. Linear regression analysis unravels the impact of mitochondrial Fenton reaction on the rise of cytoplasmic pH. **E**. Linear regression analysis exhibits a correlation between the level of mitochondrial Fenton reaction level (x-axis) and the elevation in endosomal pH (y-axis). **F**. Linear regression analysis unravels the correlation between the level of mitochondrial Fenton reaction (x-axis) and the elevation in lysosomal pH (y-axis). **G**. Comparison of the cytoplasmic pH elevation across three groups. Statistical significance was determined by Wilcoxon rank-sum test. **H**. Linear regression analysis demonstrates a relationship between the level of mitochondrial Fenton reaction (x-axis) and tau fibril formation (y-axis). **I**. Assessment of the relative contributions of seven metabolic reprogramming factors to the mitigation of Fenton reactions during the progression from MCI to AD. Samples from 339 cases (MCI and advanced AD) were ranked by their number of upregulated genes relative to controls. The x-axis represents 30 consecutive sliding windows (window size = 30, step size = 10) across this ranked order. The y-axis shows the level of mitochondrial Fenton reaction (solid line) and the individual contributions of seven balancing factors (dashed lines) that maintain mitochondrial pH stability. UCP: uncoupling protein.

We examined the expression of relevant pH-related enzyme and importer genes, namely GLS, uncoupled protein (UCP) transporters, and a few acid-loading transporters, such as *SLC4A3* and *ATP2B1* (**Figure 3**A–D; Table S3).

It should be noted that (1) GLS catalyzed the reaction:


(3) $$L-\text{glutamine}+H_{2}O\to L-\text{glutamate}+{\text{NH}}_{3}+H^{+}$$

with its gene expression being upregulated by 0.2- and 2.3-fold in MCI and AD samples over controls (Figure S2A), respectively; both the glutamine importer and the exporter genes were strongly correlated with the expression of GLS in MCI and AD tissues (Figure S3); (2) UCPs, such as ATP synthase, leaked H^+^ into the mitochondrial matrix, and converted the H^+^-potential energy to heat rather than ATP [31], which was strongly correlated with the mitochondrial Fenton reactions in AD (Figure S2B and C); and (3) *SLC4A3* exchanged HC$O_{3}^{-}$ (outwards) for Cl^−^ (inwards), while *ATP2B1* contributed to introcelluar acidification by producing H^+^ per reaction [Ca^2+^(in) + ATP + $H_{2}$O → Ca^2+^(out) + ADP + phosphate + H^+^] [31] (Figure S2D).

#### pH increases in other organelles

We noted that cytosol, endosomes, and lysosomes also showed increased pH, based on their respective marker genes. Specifically, the cytosolic pH was significantly increased in AD samples with $p^{A}$-value = 0.032, while no change was observed in MCIs, consistent with the literature [5]. The same was also observed in the endosome lumen with significant pH increases in AD (Figure S4A) and less significant increases in MCI (Figure S4A), as well as in lysosome with more significant increases in AD and less significant increases in MCI  (Figure S4B).

Strong correlations were detected between the increased level of mitochondrial Fenton reactions and the increase in pH-marker genes for cytosol, endosome, and lysosome, respectively, in both MCI and AD (for all three groups, $p^{M}$-value and $p^{A}$-value < 0.05; Figure 3D–G). Furthermore, high correlations were detected between the increased mitochondrial pH and pH in each of these three compartments in both MCI and AD patients (similarly, for all three groups, $p^{M}$ value and $p^{A}$-value < 0.05; Figure S4C–E). These findings are consistent with published studies reporting that both lysosome and endosome have elevated pH in AD samples, and that acidification of these organelles can alleviate AD symptoms [21,22].

This provides strong evidence for a cell-level mechanism that maintains pH homeostasis across different organelles [21,22], indicating that increased mitochondrial pH leads to pH increase in lysosome, endosome, and cytosol by redistributing protons across multiple compartments to share the burden of alkaline stress.

#### Formation of NFTs

The basic units of NFTs are tau monomers that are driven away from their originally bound microtubules by hyperphosphorylation, which has been widely reported [4]. It is noteworthy that phosphorylation is an acidifying reaction, as shown below:


(4) $${\text{ATP}}^{4–}+H_{2}O\to {\text{ADP}}^{3–}+H_{2}{\text{PO}}_{4}{\;}^{–}\Leftrightarrow {\text{ADP}}^{3–}+{\text{HPO}}_{4}{\;}^{2–}+H^{+}$$

In addition, the tau protein was alkaline as it contained 10 alkaline and 3 acidic amino acids, as highlighted in Figure S5A and B. Interestingly, the published structure of a tau filament consists of two tau monomers stacked on top of each other, along with an RNA inserted into each of the two clefts (Figure S5C). The binding between each negatively charged RNA and the six positively charged alkaline amino acids blocked the side chains of the alkaline amino acids from exposure to the solvent. As shown in Figure S5D and E, the higher the stack was, the more alkaline amino acids were blocked from being exposed to the solvent, hence reducing the alkalinity of the tau monomers.

We therefore propose that the tau fibril formation is induced to prevent pH from becoming overly alkaline, serving as a protective mechanism, at least during the early phase of disease development. We then conducted correlation analyses between Fenton reaction levels and NFT formation. A strong correlation was observed, as shown in Figure 3H, providing additional support for our model prediction.

#### Levels of contribution to keeping mitochondrial pH stability

To determine whether all these acidification processes may be relevant to the mitochondrial Fenton reactions, we conducted a regression analysis of the predicted Fenton reaction levels (*i.e.*, the production rate of $OH^{-}$) against all the above acid-producing events and importers, using the regression models shown below:


$$\begin{array}{c} In\;\mathrm{MCI}:Y=0.21X_{1}+\;0.16X_{2}+0.28X_{3}+0.25X_{4}\\ +0.19X_{5}+0.29X_{6},\;\mathrm{with}\;R^{2}=0.91\\ In\;\mathrm{AD}:Y=0.27X_{1}+\;0.28X_{2}+0.15X_{3}+0.26X_{4}\\ -0.002X_{5}+0.1X_{6},\;\mathrm{with}\;R^{2}=0.93 \end{array}$$

where Y is for the level of mitochondrial Fenton reaction, *X*_1_ to $X_{6}$ represent the combined expression of the acid-loading transporters, the expression of GLS, the combined expression of UCPs, the level of tau fibril formation, and the pH increase in cytosol and endosome, respectively.

Based on these findings, we predict that all six acidifying processes are induced to maintain mitochondrial pH within the normal range, thereby keeping the host cell alive. To examine the contribution of the six factors, we conducted a BIC-based analysis [37], with the results presented in **Table 3**. We conclude, based on these results, that in MCI samples, UCPs and tau fibril formation are the major contributors to maintaining intracellular pH stability, whereas in AD samples, GLS and tau fibril formation are the main contributors.

**Table 3:** Bayesian information criteria for acidification processes maintaining mitochondrial pH

Group	Acid-loading transporter	GLS	UCP	Tau fiber formation	Cytosolic pH	Endosomal pH
MCI	−2.88	−3.1	−0.58	0.89	−2.41	−2.84
AD	−0.90	2.61	−3.51	5.07	−5.26	−4.86

*Note*: GLS represents the contribution of glutaminase in mitigating mitochondrial Fenton reactions. UCPs indicate the contribution of uncoupling proteins. Cytosolic pH reflects the contribution of intracellular pH elevation, while endosomal pH represents the contribution of endosomal pH elevation.

#### Levels of alkalization and acidifying responses with the progression of the disease

To understand how the level of the Fenton reaction and its acidifying response change with disease progression, we sorted all examined AD samples by the number of upregulated genes relative to controls as an approximation of disease progression. For the ordered samples, we used a sliding window of 40 samples and displayed the levels of Fenton reactions and contributions by each of the seven balancing factors, as shown in Figure 3I and Table S4.

We noted that GLS did not play a major role initially, but it significantly increased over time in MCI samples. Throughout AD samples, a significant elevation in the Fenton-reaction level was observed, with GLS, an increase in cytosolic pH, and tau fibril formation as the major contributors.

### Fenton reactions drive neuronal hyperexcitability and extracellular acidification

We investigated how the overproduction and extracellular release of glutamate may contribute to the massive neuronal death in AD patients, given that glutamate is an excitatory neurotransmitter and hyperexcitability is a key phenotype of AD [14] and possibly a key cause of massive neuronal death [38].

**Figure 4:**
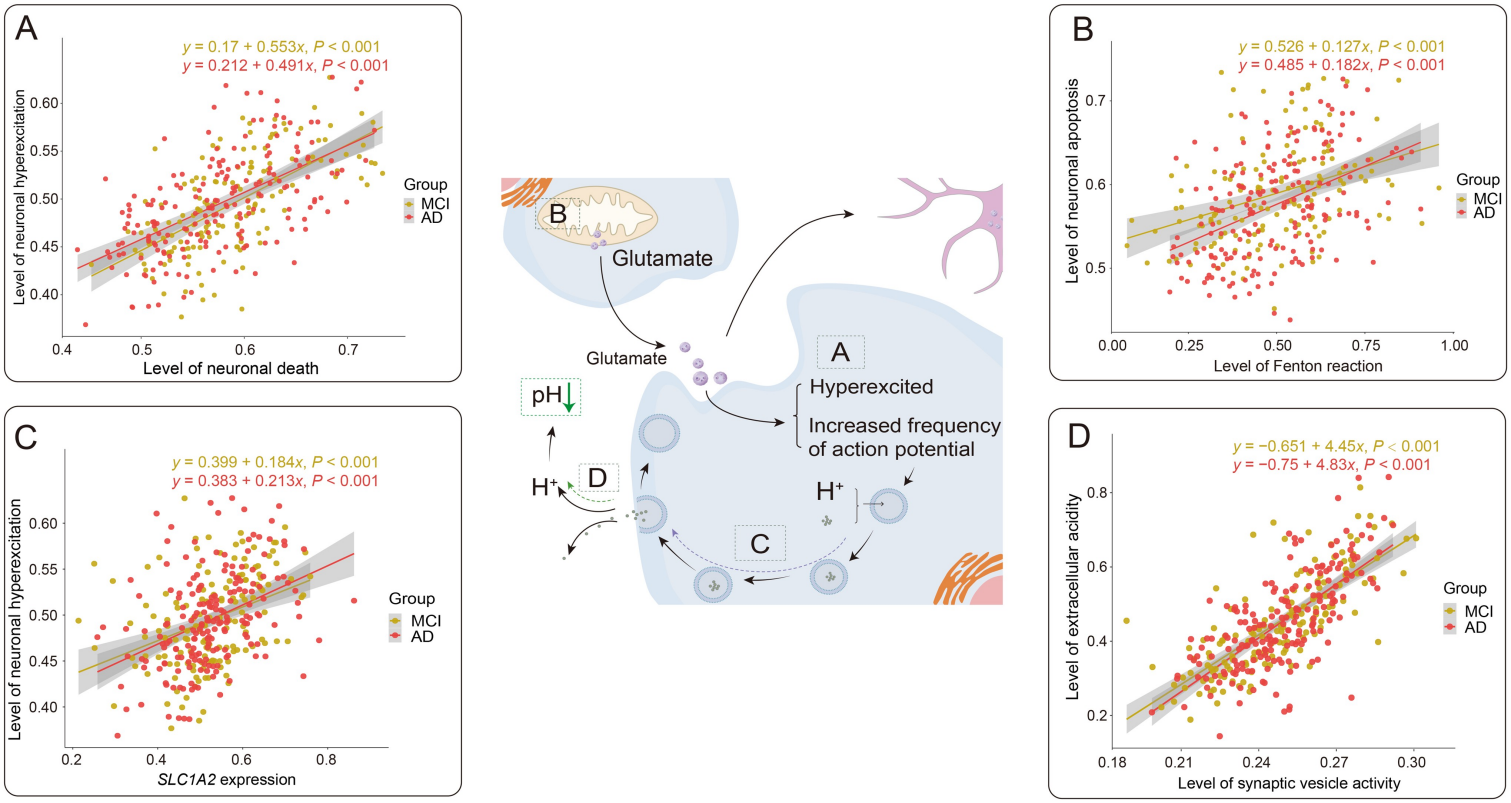
Hyperexcitability and extracellular acidity are results of mitochondrial Fenton reactions **A**. Linear regression analysis establishes a correlation between levels of neuronal death (x-axis) and neuronal hyperexcitation (y-axis). **B**. Linear regression analysis shows relationship between the level of mitochondrial Fenton reaction (x-axis) and the level of neuronal apoptosis (y-axis). **C**. Linear regression analysis shows a relationship between *SLC1A2* expression (x-axis) and neuronal hyperexcitability (y-axis). **D**. Linear regression analysis builds the relationship between the level of synaptic vesicle activity and the level of extracellular acidity.

Our analyses revealed that the level of apoptosis in AD samples strongly and positively correlated with hyperexcitability and mitochondrial levels of Fenton reaction (**Figure 4**A and B), suggesting possible connections among mitochondrial Fenton reactions, hyperexcitability, and massive neuronal death.

We observed that (1) *SLC1A2*, a transporter responsible for > 90% of CNS glutamate uptake [39], showed strong and positive correlations with neuronal hyperexcitation in AD groups (Figure 4C); (2) marker genes for neuronal hyperexcitability were considerably upregulated (Figure S6A); (3) the expression of transporter *VGLUT*, responsible for loading glutamates into synaptic vesicles, was significantly positively correlated with neuron hyperexcitation (Figure S6B); (4) the expression of genes involved in releasing synaptic vesicles was upregulated and strongly correlated with the extracellular acidity level in AD samples (Figure 4D, Figure S7A); and (5) the expression of *ASIC2* or *ASIC3*, the marker genes for extracellular acidity, was markedly upregulated in AD samples (Figure S7B), which were strongly correlated with apoptosis (Figure S7C), establishing a causal cascade from neuronal hyperexcitability to neuronal apoptosis.

**Figure 5:**
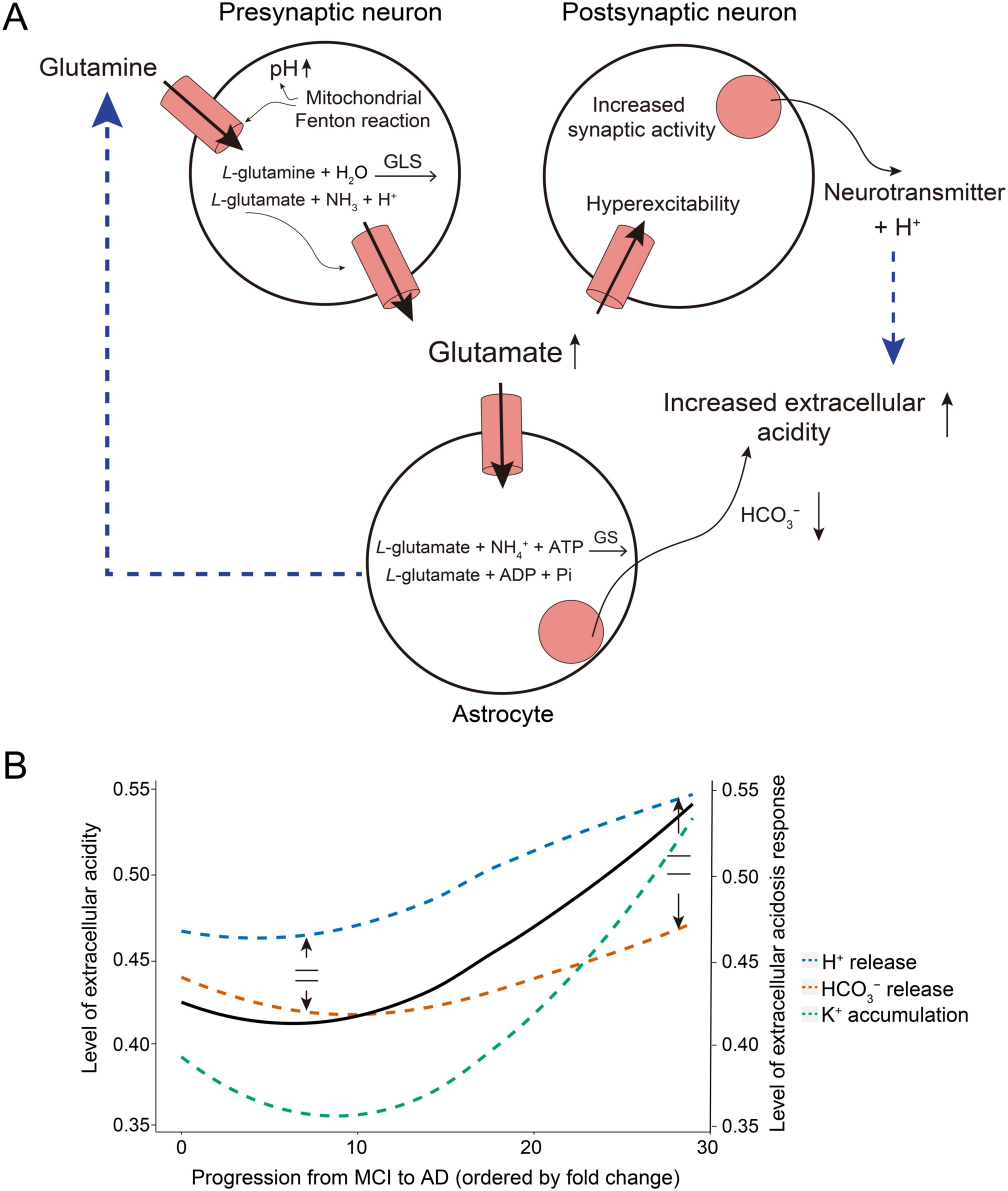
Schematic illustration for a series of events starting from overproduction of glutamate, needed for keeping intracellular pH stable and hence lifesaving, to extracellular acidity and neuronal death **A**. The key insight gained here is that the excessive production and extracellular release of glutamate lead to a cascade of effects: neuronal hyperexcitability, continuous extracellular acidity, and ultimately, neuronal death, contributing to increased extracellular acidity. **B**. Samples from 339 cases (MCI and advanced AD) were ranked by their number of upregulated genes relative to controls. The x-axis represents 30 consecutive sliding windows (window size = 30, step size = 10) across this ranked order. The y-axis is for the level of extracellular acidity (solid line) and the levels of synaptic H^+^ release, ${\mathrm{HCO}}_{3}^{-}$ release, and K^+^ accumulation (dashed lines) under the simulation of differential equations. The difference between the two arrows indicates the net level of extracellular acidity.

By integrating these statistical data and the underlying chemistry relationships, we predicted that the overproduction and release of glutamate into the extracellular space led to the hyperexcitability of neighboring neurons and over-release of H^+^-rich synaptic vesicles, which might result in persistent extracellular acidification and ultimately neuronal death. **Figure 5**A summarizes these relationships.

To close this logic loop, we need to gain a more detailed understanding of how glutamate overproduction leads to hyperexcitability and how increased release of H^+^-rich synaptic vesicles results in extracellular acidosis.

It has been suggested that hyperexcitability in AD samples results from reduced extracellular glutamate clearance by local astrocytes [38], as a reduced level of extracellular Na^+^ proportionally decreases the rate of glutamate clearance (Figure S8A) [40]. To check this, we obtained the following analysis results. First, extracellular acidity could repress Na^+^/K^+^ exchanger (Figure S8B), which resulted in an increased level of intracellular Na^+^ and a reduced level of extracellular Na^+^ [41]. Second, the relative expression level for glutamine synthetase (GS), which catalyzes the reaction to convert the glutamates (recollected by astrocytes via the glutamate-clearance process) to glutamine, was considerably lower than that for GLS (Figure S8C) in AD samples *vs*. in controls, providing strong evidence that there is an accumulation of extracellular glutamates in AD patients.

Regarding extracellular acidification, it is noteworthy that under normal conditions, H^+^ released by synaptic vesicles is neutralized by bicarbonates (${\mathrm{HCO}}_{3}^{-}$) released at the same rates by the neighboring astrocytes [26], to keep the extracellular pH stable:


(5) $${\text{HCO}}_{3}^{-}+H^{+}\Leftrightarrow H_{2}\text{CO}3\Leftrightarrow H_{2}O+{\text{CO}}_{2}$$

where CO_2_ is released from the brain, driving the neutralization reaction. It is well-known that an increased extracellular concentration of K^+^, as a result of repressed activity of the Na^+^/K^+^ exchanger, can cause astrocyte depolarization and subsequent repression of ${\mathrm{HCO}}_{3}^{-}\;$export [26]. This reduction in ${\mathrm{HCO}}_{3}^{-}$ release elevates extracellular pH, which in turn further inhibits the activity of the Na^+^/K^+^ exchanger, leading to additional extracellular K^+^ accumulation. This positive feedback loop drives the progression of the extracellular acidification, acidosis, and ultimately neuronal death.

Our data strongly supported these analyses, which could be characterized by the following differential equations and are depicted in Figure 5B and Figure S8D–F:


(6) $$\frac{d\mathrm{pH}}{dt}=([H^{+}]-[{\mathrm{HCO}}_{3}^{-}])$$

(7) $$\frac{d[H^{+}]}{dt}=-k\cdot [H^{+}]\cdot [{\mathrm{HCO}}_{3}^{-}]+([H^{+}]\cdot \mathrm{pH})\;$$

(8) $$\frac{d[{\mathrm{HCO}}_{3}^{-}]}{dt}=-k\cdot [H^{+}]\cdot [{\mathrm{HCO}}_{3}^{-}]+([{\mathrm{HCO}}_{3}^{-}]\cdot (1-\mathrm{pH}))$$

(9) $$\frac{d[K^{+}]}{dt}=[K^{+}]\cdot \mathrm{pH}$$

where pH is the level of extracellular acidity, $[H^{+}]\;$is the level of $H^{+}$ release by the synaptic vesicle, $\left[{\mathrm{HCO}}_{3}^{-}\right]$ is the level of ${\mathrm{HCO}}_{3}^{-}$ released by astrocytes, $[K^{+}]\;$is the level of extracellular $K^{+}$, and *k* represents the chemical equilibrium constant for the reaction between $H^{+}\;$and$\;{\mathrm{HCO}}_{3}^{-}$. The interactions among $\left[K^{+}\right]$, $\left[H^{+}\right]$, and $\left[{\mathrm{HCO}}_{3}^{-}\right]$ are presumed to follow a linear relationship.

It is noteworthy that in comparison to MCI samples, AD samples showed increased intracellular alkalosis and more extensive neuronal death, as evidenced by the more pronounced upregulation of marker genes for intracellular alkalinity and neuronal death in AD than in MCI *vs.* control samples (Figure S9).

### Extracellular acidosis *vs*. Aβ formation

**Figure 6:**
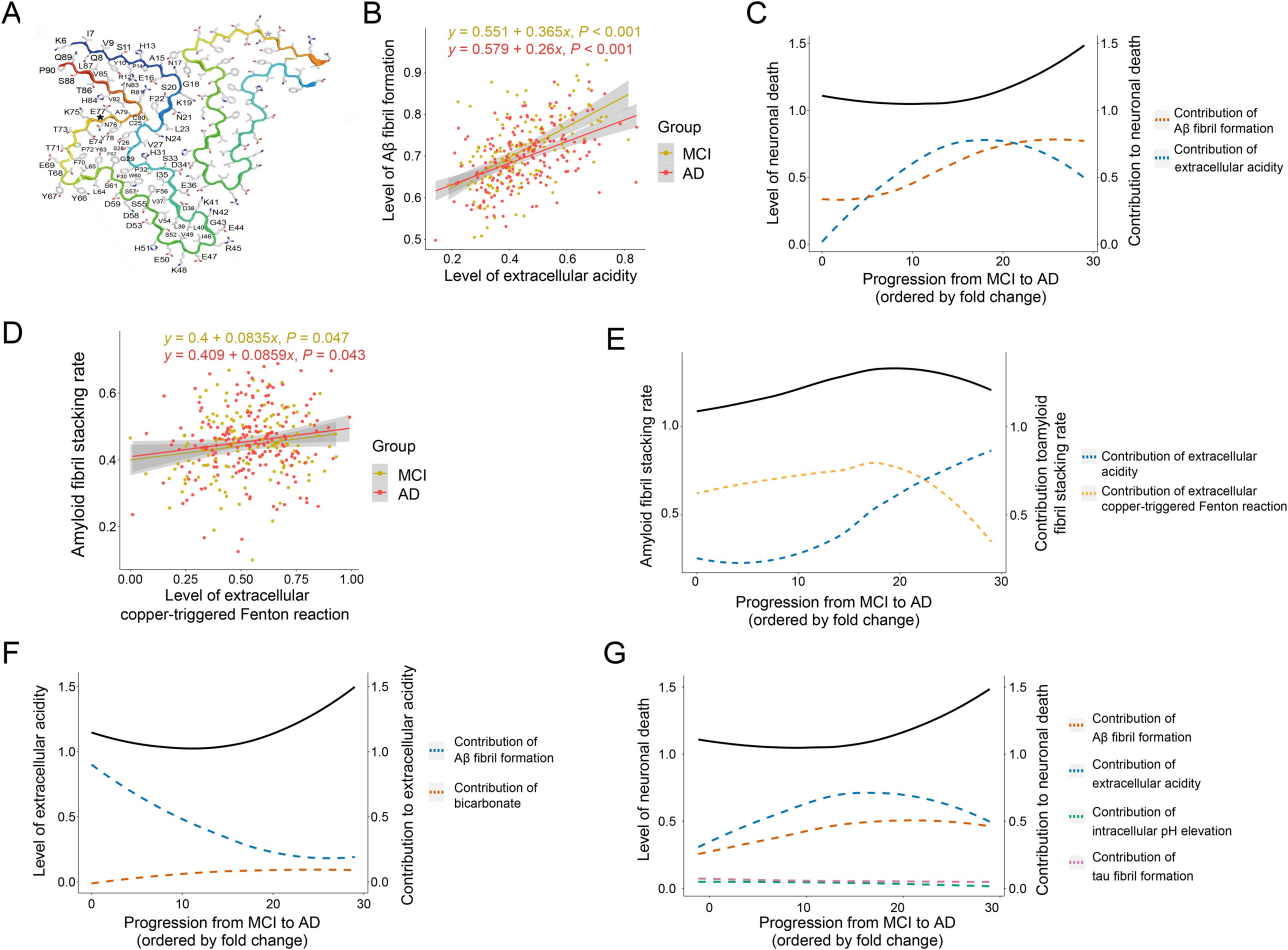
Relationship between amyloid formation and extracellular acidity **A**. A single layer of amyloid structure composed of two Aβ monomers. **B**. Linear regression analysis depicts the association between extracellular acidity (x-axis) and Aβ fibril formation level (y-axis). **C**. Contribution of Aβ fibril formation and extracellular acidity to neuronal death and the effect of extracellular acidity on amyloid formation along the disease progression. **D**. Linear regression analysis illustrates the relationship between the extracellular copper-mediated Fenton reaction and the amyloid fibril stacking rate (y-axis). **E**. Contributions of extracellular acidity and the extracellular copper-related Fenton reaction to promote the amyloid fibril stacking rate. **F**. Effects of Aβ fibril formation and reduced bicarbonate release on extracellular acidity during disease progression. **G**. Impact of four key factors — Aβ formation, tau fibril formation, extracellular acidity, and intracellular pH elevation – on neuronal apoptosis throughout disease progression. Aβ, amyloid-beta.

To understand how Aβ plaques contribute to the development of AD, as was done earlier for tau fibers, we examined a single layer of the experimentally determined stacked structure consisting of Aβ dimers (**Figure 6**A) [42]. We noted that each monomer contained five acidic and three alkaline amino acids, with side chains pointing into the solution. Interestingly, a recent study has shown that stacking of the Aβ dimers on top of each other makes the complex structure more basic than the Aβ dimer; the more dimers stacked on top of each other, the more basic the complex structure becomes [42], revealing that the acidic sidechains are largely buried inside preventing them from being exposed to the solvent, while the basic ones are not, making the overall structure more basic than the collection of individual Aβ dimers. Hence, we propose the following hypothesis that the formation of a stacked structure of Aβ dimers may slow the extracellular acidification process.

To further examine this fundamentally novel insight, which is the opposite of the mainstream view of the Aβ fibril roles, we conducted correlation analyses between Aβ fibril formation and extracellular acidification, as well as neuronal cell death. We found that (1) the formation of Aβ fibrils served a beneficial role by preventing excessive extracellular acidification in the early AD samples (Figure 6B). This may help explain why medicines targeting at Aβ fibril formation have failed to slow the disease development [43]. (2) An increasingly stronger correlation with neuronal death was observed with the disease progression, suggesting that aggregated Aβ structures (Figure 6C) increasingly contribute to neurotoxicity, potentially by impairing cell–cell communication, consistent with previous reports [44].

#### Alkalizing responses to extracellular acidosis with the progression of the disease

To understand the possible drivers of the formation of the stacking structures of the Aβ dimers, we assessed the possibility of Fenton reactions in the extracellular space, noting that ·OHs produced by Fenton reactions are key drivers for aggregation of the proteins/peptides [45]. The extracellular copper-triggered Fenton reactions significantly promoted the amyloid fibril stacking rate ($p^{A}$-value < 0.05), as shown in Figure 6D. Further BIC analyses revealed that during the early stages, the amyloid fibril stacking rate was primarily driven by the Fenton reaction triggered by extracellular copper ions, while in later stages, extracellular acidosis became the main driver (Figure 6E). We conducted correlation analyses between extracellular acidosis and several activities with the progression of the AD (see Materials and methods), with the results shown in Figure 6F.

As depicted in Figure 6G, while amyloids’ role in mitigating extracellular acidification gradually declined with disease progression, bicarbonate-mediated acid alleviation remained relatively constant throughout. In the advanced stage, the concerted efforts of both amyloid formation and bicarbonate ultimately proved insufficient against the escalating levels of acidification.

### Overall model

By integrating our new findings, we constructed a model describing how persistent mitochondrial Fenton reactions, driven by chronic inflammation and elevated iron metabolism in mitochondria, induce continuous mitochondrial alkalization. This alkalization, in turn, triggered multiple acidifying reprogrammed metabolisms that support cell survival (Figure 1). Among them, the most prominent was $H^{+}\;$production via enzyme-mediated conversion of glutamine to glutamate. This led to overproduction and extracellular release of glutamates, driving the hyperexcitability of the postsynaptic neurons and increased release of $H^{+}$-rich synaptic vesicles . Meanwhile, due to the acidic extracellular space, neighboring astrocytes were unable to match this release with sufficient bicarbonate neutralization, resulting in persistent extracellular acidification and neuronal death. We predict that some of the well-studied AD-associated events, such as the formation of Aβ plaques and tau neurofibrillary tangles, are host cell responses to persistent extracellular acidification and to intracellular alkalization, respectively, thereby serving protective roles for cell survival. Figure 1 depicts these key events and their relationships.

To determine the predominant contributors to neuronal death among key disease-associated changes — including extracellular acidification, intracellular alkalization, Aβ formation, and TNF development — we performed a regression analysis of the neuronal death against these four factors, with details provided in Figure 6E and Table S5. The results indicate that the major factors in causing neuronal death are extracellular acidification and Aβ amyloids as the first and second contributors throughout disease development, while contributions by intracellular alkalization and NFTs towards cell death are relatively minor. It is noteworthy that the formation of Aβ amyloids was driven by extracellular acidification, strongly suggesting that this should be a primary target for future AD drug design.

Eleven of the 17 predicted relationships (64.7%) in the model have direct literature support as depicted in Figure 1. In addition, there is literature support for the overall idea of our model: the glutamate-induced mitochondrial matrix acidification abrogated mitochondrial matrix pH gradient [46]. A majority of the protons resulting in extracellular acidification in brain are believed to originate from neurotransmitter release [6]. An acidic environment promotes the formation of amyloid plaques [47]. Continuous treatment of the Aβ amyloids at a physiologic dose results in impaired neuronal firing without significant cell death [4].

Our analyses also unveiled key distinctions between MCI and AD patients: (1) in individuals with AD, immune cells such as astrocytes and microglia exhibited significantly increased activities compared to those with MCI (*P* < 0.05); (2) action potential activity was notably decreased in AD *vs*. MCI patients; and (3) late-stage intracellular alkalization contributed to AD cell death, albeit to a considerably lesser extent than extracellular acidification.

To further validate the reliability of our model, we applied causal inference [71] to all predicted relationships (see Materials and methods). Overall, our analyses revealed that neither additional random causes nor placebo effects influenced the predicted causal relationships. For example, for the predicted causal relationship between extracellular acidification and Aβ plaque formation, the average treatment effect (ATE) was 0.31905. Our refutation tests showed that (1) introduction of a random cause produced an estimated effect of 0.3192 (*P* = 0.98), indicating no impact; and (2) when the effect variable was replaced by a placebo, the effect dropped to nearly zero at 0.00311.

## Discussion

AD is a very complex disease with numerous clinical phenotypes. Because of the complexity, the state-of-the-art understanding of the key drivers of the disease remains elusive. The consequence is that none of the medicines designed to treat the disease has shown any effect on slowing disease progression — the reason is obvious, the selected target is not the driver of the disease. Through careful analysis and modeling of pH stressors, guided by transcriptomic data of AD at both early and advanced stages, we developed a system-level, dynamic model for the disease, which is highly consistent with the available transcriptomic data and explains numerous unanswered questions about AD. To the best of our knowledge, this is the first such model in the world. While there is yet no experimental data directly designed to validate the model partially because the existing animal models tend to be overly simple to truly capture the essence of the disease, it provides a biologically sound and highly self-consistent framework for causal relationships among all the major characteristics of AD, with the majority of the predicted causal relationships supported by published studies.

It is known that persistent cellular pH imbalances can have profound impacts on the overall cellular chemistry and biology [21,31,35,48]. The backbone of our model is a stress-adaptation model, focusing on how cells alter their metabolisms in response to persistent production of OH^−^ for cellular survival, and how stress-induced reprogrammed metabolisms not optimized over a long evolutionary history can give rise to abnormal cellular behaviors. Therefore, a unique feature of our model is that it studies the disease at the chemistry-balance level, particularly pH imbalances.

The model focuses on two key characteristics of ADs, intracellular alkalosis and extracellular acidosis, which have long been reported [5,6,49]. The starting point of the model is persistent inflammation, coupled with local iron accumulation, leading to persistent disruption of the intracellular pH homeostasis. Multiple acidifying reprogrammed metabolisms are induced to adapt to this life-threatening stressor. It is one of the reprogrammed metabolisms, namely persistent production of glutamate and $H^{+}$ via hydrolysis of glutamine, which gives rise to the overproduction and release of proton-rich synaptic vesicles, gradually resulting in extracellular acidosis, representing the key reason for massive neuronal death.

In our model, the formation of tau tangles is induced to mitigate intracellular alkalosis, while the aggregation of Aβ plaques alleviates extracellular acidosis in the early phase of the disease. This protective role is supported by studies showing that hyperphosphorylated tau can attenuate ER stress-induced apoptosis [50] and that fibrillar Aβ plaques reduce neurotoxicity and enhance cell viability under acidic conditions [47,51]. It is noteworthy that persistent intracellular alkalosis cannot be resolved via proton transporters in a sustainable manner, since this would either violate the electroneutrality of a cell or the intracellular homeostasis of some other ions if the proton transporter is not electrogenic [52]. Furthermore, our analyses revealed that lactic acid did not contribute to the extracellular acidosis as suggested by the published studies [6], as detailed in Table S6.

The overall model is self-consistent and highly consistent with published studies [5,14,18,21–23,26–28] and hence provides a highly useful framework for more in-depth studies of AD mechanisms and treatment. Given its richness, the model offers multiple entry points for researchers — particularly experimentalists — to investigate specific issues while understanding how those issues fit within the broader context of disease progression, potentially accelerating advances in the field.

Several previous studies have implicated intracellular alkalization, extracellular acidification, and iron accumulation in AD [18,30,49], but they did not examine their causal relationships with massive neuronal cell death that defines the characteristic of the disease.

### Sex differences in AD

It is well established that the occurrence rate of AD is higher in women than in men [53]. Our current model suggests that elevated iron levels in mitochondria are a key inducer of the disease, which would seemingly predict a higher AD rate in men, given that total body iron levels are typically higher in males. To understand this inconsistency, we re-analyzed our data separately for male and female patients. Very interestingly, we noted that in female patients, not only the iron level but also the mitochondrial copper level contributed to mitochondrial Fenton reactions (Figure S11). These findings offer a potential explanation for the observed sex disparity.

## Materials and methods

### Transcript assembly, quantification, and analyses

A computational pipeline was implemented to assemble strand-specific RNA-seq reads in the ROSMAP cohort [54] and to analyze the derived transcripts as outlined and detailed in Appendix method 1 (File S1). Specifically, high-quality RNA-seq reads were obtained by processing the BAM file data to remove low-quality reads using SOAPnuke [55]. Then, the selected reads were mapped to the reference human genome in GENCODE using HISAT2 with the default parameters [56]. StringTie was employed to assemble the transcripts using HISAT2 against the reference genome [57]. The fasta file containing all assembled transcript sequences used can be found in Appendix result 1 (File S1). The Gene Transfer Format (GTF) file in Appendix result 2 (File S1) contains the assembled transcripts with genome annotations. The Ballgown package in R was used to calculate gene expression in transcripts per million (TPMs) [58]. The complete dataset of the assembled transcripts is provided in Appendix result 3 (File S1). Transcripts with at least 1 read count per sample from at least one-fifth of the samples were kept for further analyses, while otherwise removed. Overall, the dataset consisted of 15,989 annotated genes from 146 MCI and 193 AD tissue samples (Figure S12; File S1, Appendix method 2) as the basis of our study.

The DESeq2 package in R was used to identify the differentially expressed transcripts (DETs) between a disease set and a control set [59], where a transcript with the absolute value of the fold-change at least 1.3 with a *P* < 0.05 was considered a DET in the disease *vs*. control set. The differential expression results are provided in Appendix result 4 (File S1).

All assembled transcript sequences were classified into two groups: noncoding and protein-encoding transcripts determined using a majority rule by five classifiers, namely CNCI [60], CPAT [61], CPC2 [62], CPPred [63], and PLEK [64]. All the protein-encoding transcripts were then reannotated functionally using the eggNOG-mapper against the eggNOG database (v4.5.1) [65]. Functional annotations of the assembled protein-coding transcripts are reported in Appendix result 5 (File S1).

ClusterProfiler [66], an R package, was used for pathway enrichment analyses of the selected genes against the GO Biological Processes [67] and KEGG PATHWAY database [68], using *P* < 0.05 as the cutoff. A comprehensive list of all enriched pathways analyzed in this study is available in Appendix result 6 (File S1).

### Co-expression analyses

To evaluate the correlation between the expression level of a query gene set *g* and a reference gene set *M* across a given collection of tissue samples, we employed two complementary strategies. In the GSVA-based linear regression approach, we first computed the normalized GSVA score of [69], denoted by $S_{\mathrm{gsva}}$. We then fit a linear model to explain the mean expression of gene set gg, hereafter e(g):


(10) $$e(g)=\beta_{1}S_{\mathrm{gsva}}+\beta_{0}$$

To estimate the parameters $\left\{\beta_{1},\beta_{0}\right\}$, we solved a constrained optimization problem aimed at obtaining an intercept $\beta_{0}$ of minimal absolute magnitude. In practice, this amounts to minimizing a composite objective of the form:


(11) $$\underset{\beta_{0},\beta_{1}}{\min}\;\{\sum_{i=1}^{N}\;[{e_{i}(g)-\beta_{1}S_{\mathrm{gsva},i}-\beta_{0}]}^{2}+\lambda |\beta_{0}|\}$$

where $e_{i}(g)\;$and $S_{\mathrm{gsva},i}$ respectively denote the expression of *g*, and the GSVA score in the *i*-th sample, and λ is a regularization constant (tuned to favor a small intercept). Empirically, we implemented this via a coordinate-descent algorithm that sequentially updates $\beta_{0}$ and $\beta_{1}$ to reduce the sum of squared residuals while shrinking $\beta_{0}$ toward zero.

In the principal-component (PC) regression approach, we extracted the first two principal components, PC1 and PC2, of the expression matrix defined by genes in *M*, provided that PC1 and PC2 jointly explained at least 75% of the total variance. We then fit the linear model


(12) $$e(g)=\beta_{1}PC_{1}+\beta_{2}PC_{2}+\beta_{0}$$

where e(g) denotes the mean expression of set *g*. Again, we used a similar objective to ensure that $\beta_{0}$ remains small in absolute value, and we solved for $\left\{\beta_{0},\beta_{1},\beta_{2}\right\}$ via the same coordinate-descent procedure. When PC1 and PC2 did not account for at least 75% of the variance, this regression still served to assess statistical significance, but without imposing a variance-based cutoff on the PCs. Following the parameter estimation in each strategy, we tested whether the resulting regression provided a statistically significant relationship between *g* and *M*. In addition, we employed Spearman’s rank correlation [70] and analyzed the residual plots derived from the linear regression fits to evaluate potential non-linear relationships between gene *g* and the gene set *M*. Detailed results are provided in Appendix result 7 (File S1).

### Causal inference for prediction validation

To ascertain the predicted causal relationships between gene sets, we used the causal inference library DoWhy [71] to evaluate the causal relationships between pairs of variables. We constructed causal models based on correlation analyses coupled with chemical balance relationships and estimated the causal effect of the treatment variable X on the outcome variable Y using the backdoor adjustment method. The causal effect was estimated as:


(13) $$Y=\beta_{0}+\beta_{X}X+\varepsilon$$

where $\beta_{X}X$ is the causal effect of X on Y, and ε is the error term.

To control for potential confounders, we applied the backdoor adjustment, conditioning on variables Z that block non-causal paths between X and Y, with the specific conditional variables for each evaluated pair provided in Appendix result 8 (File S1):


(14) $$P(Y|X)=\sum_{z\in Z}\;P(Y|X,Z)P(Z)$$

We then estimated the causal effect ${\hat{\beta }}_{X}$ using a linear regression:


(15) $${\hat{\beta }}_{X}=\frac{\sum_{i=1}^{n}\;(X_{i}-\bar{X})(Y_{i}-\bar{Y})}{\sum_{i=1}^{n}\;(X_{i}-\bar{X}{)}^{2}}$$

To validate the robustness of the causal effect estimate, we conducted three refutation tests: (1) In the random common cause refuter, we introduced random noise as a pseudo-confounder; the correct result was that the causal effect remained unchanged [72], indicating no sensitivity to unaccounted confounders. (2) In the placebo treatment refuter, we permuted the treatment variable X; the correct result was no significant effect [73], as a placebo treatment should not influence the outcome. And (3) In the data subset refuter, we re-estimated the causal effect on different data subsets [74]; the correct result was consistent effects across subsets, confirming the model’s stability.

Furthermore, to enhance the credibility of the causal relationships, we also employed structural equation modeling (SEM) [75] to validate the logical relations; the detailed SEM procedures are provided in Appendix method 4 (File S1), and the validation results can be found in Appendix result 9 (File S1). These tests ensured the robustness of the causal estimates.

### Ordering disease samples based on their derivation levels from normal controls

We ordered the samples of MCI and AD to mimic the progression of the disease, specifically the following disease indicators progressively becoming worse: neuronal death rate, extracellular acidity, intracellular alkalinity, Aβ formation, tau fibril formation, and oxidative stress. We modeled this issue as follows: let G be the set of marker genes for the above disease characteristics, whose expressions increase with progression of the disease. Let


(16) $$V_{i}=\sum_{g\in G}E_{i}(g\;|\;\mathrm{if}\;g\;\mathrm{is}\;\mathrm{upregulated}\;\mathrm{in}\;\mathrm{MCI}\cup \mathrm{AD})$$

where $E_{i}(g)\;$is the expression level in sample *i.* We noted that sample ordering based on {$V_{i}$}was sensitive to the definition of “upregulation”, indicating the threshold value of fold change (FC). Hence, we formulated the sample order problem by solving the following problem:


(17) $$\underset{\mathrm{FC}\in [1.1,4.0]}{\max}\;\sum_{i=1}^{\left|\mathrm{MCI}\cup \mathrm{AD}\right|}\;V_{i}$$

Subject to the following conditions: the first ∣MCI∣ samples in the ordering must contain at least γ% of the MCI samples, and the last $\frac{\left|\mathrm{MCI}|+|\mathrm{AD}\right|}{2}$ samples must contain at least γ% of the AD samples. The maximization problem was solved by evaluating all possible FC thresholds.

## Code availability

The complete dataset and relevant source codes can be accessed and downloaded from the BioCode at the National Genomics Data Center (NGDC), China National Center for Bioinformation (CNCB) (BioCode: BT007921), which are publicly accessible at https://ngdc.cncb.ac.cn/biocode/tools/BT007921.

## CRediT author statement

**Zhenyu Huang:** Resources, Conceptualization, Methodology, Investigation, Visualization, Project administration, Writing – original draft, Writing – review & editing. **Xuechen Mu:** Resources, Conceptualization, Methodology, Investigation, Visualization, Writing – original draft. **Qiufen Chen:** Resources, Visualization. **Lingli Zhong:** Resources, Visualization. **Jun Xiao:** Resources, Visualization. **Chunman Zuo:** Resources, Methodology, Visualization. **Ye Zhang:** Resources, Conceptualization, Investigation. **Bocheng Shi:** Resources, Visualization. **Yingwei Qu:** Resources, Visualization. **Renbo Tan:** Resources, Visualization. **Long Xu:** Resources, Visualization. **Renchu Guan:** Resources, Methodology. **Ying Xu:** Resources, Conceptualization, Methodology, Investigation, Visualization, Project administration, Writing – original draft, Writing – review & editing. All authors have read and approved the final manuscript.

## Competing interests

The authors have declared no competing interests.

## Supplementary material

Supplementary material is available at *Genomics, Proteomics & Bioinformatics* online (https://doi.org/10.1093/gpbjnl/qzaf087).

## Supplementary Material

qzaf087_Supplementary_Data
