# Supplementary material for: A Model for the Development of Alzheimer’s Disease
Source: Genomics Proteomics Bioinformatics. 2025 Sep 23;23(6):qzaf087. doi: 10.1093/gpbjnl/qzaf087 (PMC13365266; doi:10.1093/gpbjnl/qzaf087)
Supplement: qzaf087_Supplementary_Data [file qzaf087_supplementary_data.zip › Figure S11.pdf]

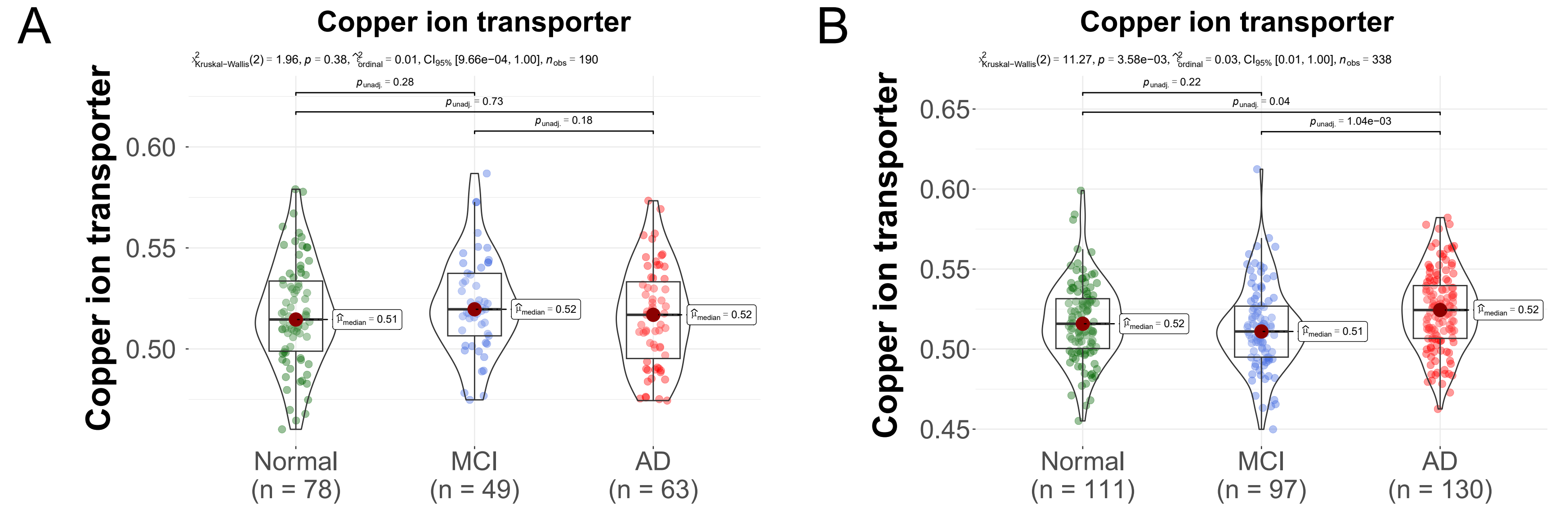
A
Copper ion transporter
$\chi^2_{\text{Kruskal-Wallis}}(2) = 1.96$, $p = 0.38$, $\hat{\epsilon}^2_{\text{ordinal}} = 0.01$, $\text{CI}_{95\%}$ [9.66e-04, 1.00], $n_{\text{obs}} = 190$
$p_{\text{unadj.}} = 0.28$
$p_{\text{unadj.}} = 0.73$
$p_{\text{unadj.}} = 0.18$
Copper ion transporter
0.60
0.55
0.50
$\hat{\mu}_{\text{median}} = 0.51$
$\hat{\mu}_{\text{median}} = 0.52$
$\hat{\mu}_{\text{median}} = 0.52$
Normal (n = 78)
MCI (n = 49)
AD (n = 63)
B
Copper ion transporter
$\chi^2_{\text{Kruskal-Wallis}}(2) = 11.27$, $p = 3.58\text{e-}03$, $\hat{\epsilon}^2_{\text{ordinal}} = 0.03$, $\text{CI}_{95\%}$ [0.01, 1.00], $n_{\text{obs}} = 338$
$p_{\text{unadj.}} = 0.22$
$p_{\text{unadj.}} = 0.04$
$p_{\text{unadj.}} = 1.04\text{e-}03$
Copper ion transporter
0.65
0.60
0.55
0.50
0.45
$\hat{\mu}_{\text{median}} = 0.52$
$\hat{\mu}_{\text{median}} = 0.51$
$\hat{\mu}_{\text{median}} = 0.52$
Normal (n = 111)
MCI (n = 97)
AD (n = 130)
