# Supplementary material for: A Model for the Development of Alzheimer’s Disease
Source: Genomics Proteomics Bioinformatics. 2025 Sep 23;23(6):qzaf087. doi: 10.1093/gpbjnl/qzaf087 (PMC13365266; doi:10.1093/gpbjnl/qzaf087)
Supplement: qzaf087_Supplementary_Data [file qzaf087_supplementary_data.zip › File S1.docx]

**File S1 Supplementary methods**

**Appendix result 1: Assembled transcript sequences**
The assembled transcript sequence data, containing all the nucleotide sequences of transcripts obtained through transcriptomic analysis.

**Appendix result 2: Corrected genome GTF annotation file**
The corrected genome GTF annotation file, which includes annotated information on genes and transcripts in the genome, used for subsequent analysis.

**Appendix result 3: Expression levels of all assembled transcripts**
Data on the expression levels of all assembled transcripts, typically presented in units such as TPM.

**Appendix result 4: Information on differentially expressed transcripts**
Detailed information on differentially expressed transcripts, including the results of differential expression analysis, statistical significance, and the upregulated/downregulated genes.

**Appendix result 5: Re-annotation of protein-coding transcript functions**
Results of the re-annotation of the functions of protein-coding transcripts, including updated functional classifications and annotations for each transcript.

**Appendix result 6: Pathway enrichment results for differentially expressed transcripts**
Pathway enrichment analysis results for differentially expressed transcripts, including the association with different pathways and the significance level of enrichment.

**Appendix result 7: Nonlinear fitting data test**
Results from the nonlinear fitting data test, providing the outcomes and parameters related to the nonlinear regression analysis performed on the data.

**Appendix result 8: Causal inference test**
Results from the causal inference test, describing the process and outcomes of inferring causal relationships from the data.

**Appendix result 9: SEM causal inference test**
Results from the Structural Equation Modeling (SEM) causal inference test, which uses SEM methods to analyze causal relationships within the data.

**Appendix result 10: Sample information**
Detailed information about the samples used in the experiment, including the source, type, quantity, and processing conditions of the samples.

**Appendix method 1: A detailed description of the AD data analysis pipeline**

A summary of the data analysis process is presented here.

**Transcript assembly**: 528 ROSMAP samples were employed for the assembly of transcripts, which were further categorized into coding and non-coding proteins. Subsequently, functional identification of the transcripts coding for coding proteins was conducted.

**Enrichment analyses**: An initial enrichment analysis was performed on all differentially expressed transcripts. Transcripts exhibiting significant regression on the first pathway defined were subjected to a second pathway enrichment. SEM (Structural Equation Modeling) was utilized to evaluate the causal association between the two enrichments (this aspect of the results is not presented in this paper, owing to early-stage data exploration).

**Pathway network assessment**: To examine the pathway network, loops linked to the GO path graph were eliminated, given its DAG graph nature with dependency relationships (this aspect of the results is not presented in this paper, owing to early-stage data exploration).

**Evaluation of gene sets**: GSVA and PCA was applied for the assessment of gene sets, while regression logic was employed to deduce associations between the target gene set. The complete code of this pipeline is now available at an ZENODO repository:

(https://drive.google.com/drive/folders/1KysxcVg9QiJK3UQ9bzem09jtNjbT9G-f).

**Appendix method 2: Data from the significantly improved transcriptomes of AD tissues**

528 samples of RNA-seq raw data of dorsolateral prefrontal cortex are retrieved from the Religious Orders Study and Memory and Aging Project (ROSMAP) cohort [1]. Of these, 146 are Mild Cognitive Impairment (MCI), 193 are Alzheimer Disease (AD), and 146 are matching control tissues, i.e., aged normal tissues, denoted by Normal. Their sample ID is summarized in Supplementary File 11. 117 samples of RNA-seq raw data from fusiform gyrus are downloaded from the GEO dataset (GSE95587) [2]. As summarized in Supplementary File S10. In addition, a total of 1283 RNA-seq samples are available from the Mount Sinai Medical Center Brain Bank (MSBB) cohort [3], including those from the frontal pole, superior temporal gyrus, para-hippocampal gyrus, and inferior frontal gyrus, also shown in Supplementary File S11.

As ROSMAP cohort holds strand-specific libraries, the detailed information about the processing of this dataset, along with their associated tools, is summarized in Table S8.

**Appendix method 3: Basic framework**

All Alzheimer's disease (AD) patients under study were found to have elevated levels of $H_{2}O_{2}$ and $O_{2}^{\cdot-}$production and local iron accumulation compared with the control tissues, based on our analyses of gene-expression data. Together they give rise to increased Fenton reactions in AD tissue cells. To estimate the levels of Fenton reactions and their impact on the intracellular pH, we consider the following chemical reactions:

$\mathrm{Fe}^{2+}+H_{2}O_{2}\to\cdot\mathrm{OH}+\mathrm{OH}^{-}+\mathrm{Fe}^{3+}(\text{ Fenton reaction)}$ （1）

$O_{2}^{\cdot-}+\mathrm{Fe}^{3+}\to\mathrm{Fe}^{2+}+O_{2}$ (2)

$\mathrm{NAD}(P)H+H^{+}+2\mathrm{Fe}^{3+}\to2\mathrm{Fe}^{2+}+\mathrm{NAD}(P)^{+}+2H^{+}$ （3）

$\mathrm{NADH}+H^{+}+2O_{2}\to2O_{2}^{\cdot-}+\mathrm{NAD}^{+}+2H^{+}$ （4）

$\mathrm{NAD}(P)^{+}+\mathrm{RH}_{2}\to\mathrm{NAD}(P)H+H^{+}+R$ （5）

Equations (2) represent the two predominant reactions responsible for the reduction of intracellular Fe³⁺ in human cells [1]. The critical superoxide-producing reaction, Equation (3), originates from two sources in AD: Complexes I and III of the mitochondrial electron transport chain (ETC) and NADH oxidases in innate immune and stromal cells, both of which are well-established. Equation (4) illustrates a broad category of reactions that reduce NADP⁺ to NADPH via a reducing agent (RA). It is important to note that hydroxide ions (OH⁻) produced by Fenton reactions may be neutralized by protons generated through some of these reactions, while others will consume protons from the pH buffer due to the absence of matching protons produced by Fenton or related reactions. This process can elevate intracellular pH levels unless neutralized by protons derived from alternative sources. Specifically, if Fe³⁺ is reduced by NAD(P)H or $O_{2}^{\cdot-}$generated by Equation (4), the OH⁻ from Equation (1) will be neutralized by protons from Equation (3) or Equation (4). Conversely, if Fe³⁺ is reduced by an exogenous $O_{2}^{\cdot-}$ or remains unreduced, the OH⁻ produced will consume a proton within the pH buffer. Exogenous superoxide refers to those formed in mitochondria that diffuse into the cytosol via the VDAC channels on the mitochondrial outer membrane [2], or those generated by NADH oxidases on the surface of immune or stromal cells, which can enter cancer cells through anion transporters [3]. This variability explains the differing net amounts of OH⁻ and ∙OH generated by Fenton reactions, adding complexity to our analyses. Overall, continuous Fenton reactions can be reformulated as follows, considering Fe is not consumed and is treated as a catalyst:

$\mathrm{RH}_{2}+2H_{2}O_{2}\to R+2\cdot\mathrm{OH}+2H_{2}O$ （6）

for the former case,

$O_{2}^{\cdot-}+H_{2}O_{2}\to\cdot\mathrm{OH}+\mathrm{OH}^{-}+O_{2}$ （7）

for the latter case.

**Appendix method 4:** **Structural equation modeling (SEM) for causal validation**
To ascertain potential causal relations between gene sets that exhibited statistical correlations, we applied a structural equation modeling (SEM) framework [4]. In this framework, each gene set is treated as a latent variable, denoted by $\eta$, capturing the underlying biological behavior. The expression values of individual genes within a given set serve as observed variables, denoted as vector $\mathbf{x}$. Conceptually, we specify a measurement equation:

$\mathbf{x}=\Lambda\eta+\boldsymbol{\epsilon},$ (8)

where $\Lambda$ is the factor-loading matrix linking the latent construct $\eta$ to its observed indicators, and $\boldsymbol{\epsilon}$ represents the measurement error. To model potential causal links between two latent variables $\eta_{i}$ and $\eta_{j}$ (corresponding to two distinct gene sets), we employ a structural equation:

$\eta_{j}=\beta_{ij}\eta_{i}+\zeta_{j}$, (9)

with $\beta_{ij}$ denoting the path coefficient (i.e., putative causal effect) of $\eta_{i}\to\eta_{j}$, and $\zeta_{j}$ capturing the residual variance and any exogenous influences. SEM then estimates these parameters $\{\Lambda,\beta_{ij}\}$ by minimizing the discrepancy between the empirical covariance matrix of the observed data and the model-implied covariance matrix.

To infer that a gene set $\eta_{i}$ causally influences another gene set $\eta_{j}$, we required the final model to attain acceptable global goodness-of-fit criteria [5]: 1. $\chi^{2}$ p-value > 0.05, indicating that the null hypothesis (no significant difference between the observed and model-implied covariance structures) cannot be rejected; 2. RMSEA < 0.08, signifying an acceptable approximation of the model to the data; and 3. CFI > 0.90, demonstrating that the proposed model achieves substantially better fit relative to a null (independence) model. The SEM validation results for 32 logical chains in our proposed network are provided in Appendix result 9 (File S1).

**References**

[1] Bennett DA, Schneider JA, Arvanitakis Z, Wilson RS. Overview and findings from the religious orders study. Curr Alzheimer Res 2012;9:628–45.

[2] Friedman BA, Srinivasan K, Ayalon G, Meilandt WJ, Lin H, Huntley MA, et al. Diverse brain myeloid expression profiles reveal distinct microglial activation states and aspects of Alzheimer’s Disease not evident in mouse models. Cell Rep 2018;22:832–47.

[3] Wang M, Beckmann ND, Roussos P, Wang E, Zhou X, Wang Q, et al. The Mount Sinai cohort of large-scale genomic, transcriptomic and proteomic data in Alzheimer’s disease. Sci Data 2018;5:180185.

[4] Hair JF, Hult GTM, Ringle CM, Sarstedt M, Danks NP, Ray S. An introduction to structural equation modeling. In: Hair JF, Hult GTM, Ringle CM, Sarstedt M, Danks NP, Ray S, editors. Partial least squares structural equation modeling (PLS-SEM) using R: a workbook. Cham: Springer International Publishing; 2021, pp.1–29.

[5] Xia Y, Yang Y. RMSEA, CFI, and TLI in structural equation modeling with ordered categorical data: the story they tell depends on the estimation methods. Behav Res Methods 2019;51:409–28.
