# Supplementary material for: A Model for the Development of Alzheimer’s Disease
Source: Genomics Proteomics Bioinformatics. 2025 Sep 23;23(6):qzaf087. doi: 10.1093/gpbjnl/qzaf087 (PMC13365266; doi:10.1093/gpbjnl/qzaf087)
Supplement: qzaf087_Supplementary_Data [file qzaf087_supplementary_data.zip › Supplementary material captions.docx]

**Supplementary material**

**File S1 Supplementary methods**

**Figure S1 Occurrence of mitochondrial Fenton reactions**

**A.** Comparison of the levels of hydroxyl radicals across three groups. The x-axis is for groups, and the y-axis for the level of hydroxyl radicals. The data are presented as median ± standard deviation (SD) for each group. **B.** Comparison of hydrogen peroxide biosynthesis levels across three groups. The violin plot displays the distribution of hydrogen peroxide biosynthesis levels for three groups (the x-axis.) The y-axis is for the level of hydrogen peroxide biosynthesis, and the width of each violin plot reflects the density of the data. **C.** The levels of superoxide anion generation among three groups. The x-axis is for the groups, and the y-axis is for the level of superoxide anion generation. **D.** Comparison of the levels of astrocyte activation across three groups. **E.** Linear regression analysis between astrocyte activation and $\left[ {\cdot O}_{2}^{-} \right]$ concentration. **F.** Linear regression analysis between astrocyte activation and [$H_{2}O_{2}]$ concentration.

**Figure S2 Acidifying reprogrammed metabolisms and importers**

**A.** Heatmaps for differential expressions of transcripts related to glutamine-to-glutamate conversion. Rows for transcripts and columns for experimental conditions. The expression level of each transcript is color-coded for each condition, with green for downregulation and red for upregulation. **B.** Comparison of the level of mitochondrial Fenton reaction among three groups. **C.** Comparison of UCP transporters among three groups. **D.** Comparison of acid-loading transporters among three groups.

**Figure S3 Expressions of GLS and impacts**

**A.** Linear regression analysis between level of EAAT and glutaminase in both MCI and advanced AD. **B.** Linear regression analysis between level of VGLUT and glutaminase in both MCI and advanced AD.

**Figure S4 Increase in pH in other organelles**

**A.** Comparison of endosome lumen acidification among three groups. **B.** Comparison of lysosome pH elevation among three groups. **C. L**inear regression analysis between level of mitochondrial Fenton reaction and endosome lumen acidification. **D.** Linear regression analysis between level of mitochondrial Fenton reaction and lysosome pH elevation. **E.** Linear regression analysis between increased cytosol pH and lysosome pH elevation.

**Figure S5 A schematic illustration of Tau fibrillary tangle**

**A.** The secondary structure of Tau peptide. **B.** The amino acid sequence of Tau, where amino acids marked in red are basic amino acids (total: 19) and those marked in blue are acidic (total: 8). **C.** A Tau homodimer, the basic unit of the Tau fibrils, with two segments of RNA molecules (blue rectangles) inserted in them. **D.** A stacked structure of the Tau homodimers on top of each other. **E.** RNA binding shields the solvent exposure of six positively charged alkaline amino acids.

**Figure S6 Correlation between the level of synaptic vesicle recycling and the vesicular glutamate transporter**

**A.** Comparison of neuron hyperexcitability among three groups. **B.** Linear regression analysis between level of VGLUT and synaptic vesicle recycling in both MCI and advanced AD.

**Figure S7 Impact of the synaptic vesicle recycling and extracellular acidity**

**A.** Comparison of synaptic vesicle recycling among three groups. **B.** Comparison of extracellular acidity among three groups. **C.** Linear regression analysis between level of extracellular acidosis and neuron death in both MCI and advanced AD.

**Figure S8 Reduced astrocytic activity *vs*. extracellular acidity and decreased clearance of extracellular glutamate**

**A.** Linear regression analysis between level of hyponatremia and glutamate importer among three groups. **B.** Linear regression analysis between level of extracellular acidity and Na^+^/K^+^ exchanger among three groups. **C.** Linear regression analysis between level of GLS and GS among three groups. **D.** A comparison was made between the rate of bicarbonate release by adjacent astrocytes and the rate of glutamate release in AD and control groups. **E.** Comparison of bicarbonate release among three groups. **F.** Comparison of Na^+^/K^+^ exchanger among three groups

**Figure S9 Neuronal death**

**Figure S10 Amyloid formation**

**Figure S11 Comparison of copper transporters between men and women in AD patients**

**A.** Comparative analysis of copper transporters among three groups in men with AD. **B.** Comparative analysis of copper transporters among three groups in women with AD.

**Figure S12 False positive and false negative rates of isoforms in the current transcriptomic data sets**

**A.** Proportions (y-axis) of the differentially expressed transcripts (DETs) for MCI and AAD samples in ROSMAP and MSBB cohorts (x-axis). **B.** Statistics of the number of protein-coding differential expression transcripts in GSE95587 and the other brain regions in MSBB cohort. **C.** False positives in protein-coding transcripts. The overlap refers to transcript names in both GENCODE and StringTie annotations. **D.** False negatives in protein-coding transcripts occur when there is an overlap between transcript names in both the GENCODE database and StringTie annotation, representing that the same transcript name is associated with the encoding of a different protein. **E.** Proportions (y-axis) of differentially expressed protein-coding transcripts (DETs) in GENCODE and StringTie (x-axis).

**Table S1 Protein degradation and Fenton reaction model**

**Table S2 Marker genes for mitochondrial Fenton reaction**

**Table S3 Acid-loading transporters and mitochondrial pH**

**Table S4 Fenton reaction levels and disease progression**

**Table S5 Neuronal death and contributing factors**

**Table S6 Differential expression of lactate-enzymes and transporters**

**Table S7 Additional relevant genes**

**Table S8 Software and tools used in the study**
