# Supplementary material for: A Model for the Development of Alzheimer’s Disease
Source: Genomics Proteomics Bioinformatics. 2025 Sep 23;23(6):qzaf087. doi: 10.1093/gpbjnl/qzaf087 (PMC13365266; doi:10.1093/gpbjnl/qzaf087)
Supplement: qzaf087_Supplementary_Data [file qzaf087_supplementary_data.zip › Table S1.docx]

**Table S1 Nonlinear regression analysis of hydroxyl radical**

| **Group** | **H_2_O_2_**  **Coef** | **·O_2_^-^**  **Coef** | **MIS**  **Coef** | **Averaged R^2^ value** | ***P* value** |
| --- | --- | --- | --- | --- | --- |
| MCI | 0.19 | 0.26 | 1.76 | 0.9 | < 0.001 |
| AD | 0.23 | 0.13 | 1.84 | 0.9 | < 0.001 |
