# Supplementary material for: A Model for the Development of Alzheimer’s Disease
Source: Genomics Proteomics Bioinformatics. 2025 Sep 23;23(6):qzaf087. doi: 10.1093/gpbjnl/qzaf087 (PMC13365266; doi:10.1093/gpbjnl/qzaf087)
Supplement: qzaf087_Supplementary_Data [file qzaf087_supplementary_data.zip › Table S2.docx]

**Table S2 Fenton reaction four quantities gene list**

| **Cellular state or biological activity** | **Marker genes** |
| --- | --- |
| Mitochondrial iron sulfur clustering synthesis genes | *GLRX5, HSPA9, GLRX2, GLRX3, MOCS1, LYRM4, FDXR, BCS1L, CIAPIN1, PRIM2, NARFL, IREB2, ISCU, ETFDH, REV3L, NFS1, NTHL1, ABCE1, ELP3, RFESD, NFU1, NUBPL, UROS, CIAO1, PPOX, ST13, UROD, TST, RSAD2, RSAD1, HSCB, SDHAF1, XDH, CISD2, CISD3, CISD1, ISCA1, ISCA2, ACO1, ACO2, UQCRFS1, FDX1, NUBP2, NUBP1, AIFM3, CDKAL1, GRPEL1, GRPEL2, ALAD, MUTYH, POLE, BRIP1, FECH, HMBS, NDUFV2, NDUFV1, ALAS1, POLD1, ALAS2, FAM96A, FAM96B, CPOX, TYW1, PPAT, FDX1L, CDK5RAP1, POLA1, UQCR11, ERCC2, DNA2, DDX11, ABCB7, LIAS, SDHA, FXN, RTEL1, BOLA3, DPYD, CYC1, NDUFS1, NDUFS2, NDUFS3, NDUFS4, NDUFS6, NDUFS7, NDUFS8, SDHC, SDHB, SDHD* |
| Superoxide anion generation | *CRP, CYBA, AGT, EGFR, F2RL1, FPR2, AATF, GNAI2, GNAI3, GSTP1, ITGAM, ITGB2, MAPT, ACP5, PON3, PRKCD, CD177, CLEC7A, SOD1, SYK, TGFB1, TYROBP* |
| Hydrogen peroxide biosynthetic process | *MTCO2P12, CTNS, FYN, MPV17L, DUOXA2, MT-CO2, STAT3, ZNF205, DUOXA1* |
| Proteasome-mediated ubiquitin-dependent protein catabolic process | *AGAP3, AKIRIN2, AKT1, AMFR, AMN1, ANAPC1, ANAPC4, ANAPC5, ANAPC10, ANAPC11, ANKIB1, ANKRD9, ANKZF1, APC, APC2, APPBP2, ARAF, ARIH1, ARIH2, ARMC8, ARRB1, ARRB2, ASB2, ASCC2, ASCC3, ATG7, ATXN3, AUP1, AURKA, AXIN1, AXIN2, BAG2, BAG5, BAG6, BBS7, BCAP31, BFAR, BIRC2, BMAL1, BTRC, CALR, CALR3, CALR4, CAML, CANX, CAV1, CBFA2T3, CCAR2, CCDC47, CCNF, CD2AP, CDC20, CDC20B, CDC23, CDC26, CDC34, CDC34B, CHFR, CLEC16A, CLGN, CLOCK, CLU, COMMD1, COP1, CRBN, CSNK1A1, CSNK1D, CSNK1E, CTNNB1, CUL1, CUL2, CUL3, CUL4A, CUL5, DAB2, DCAF11, DCAF12, DDA1, DDB1, DDIT3, DDRGK1, DERL1, DERL2, DERL3, DESI1, DET1, DMAC2, DNAJB2, DNAJB9, DNAJC10, DTL, E330034G19RIK, ECPAS, ECRG4, EDEM1, EDEM2, EDEM3, EIF2AK3, EIF3H, EPM2A, ERCC8, ERLEC1, ERLIN1, ERLIN2, FAF1, FAF2, FBXL2, FBXL3, FBXL4, FBXL5, FBXL6, FBXL7, FBXL8, FBXL9, FBXL12, FBXL13, FBXL14, FBXL15, FBXL16, FBXL17, FBXL18, FBXL19, FBXL20, FBXL21, FBXL22, FBXO2, FBXO3, FBXO4, FBXO6, FBXO9, FBXO17, FBXO22, FBXO27, FBXO31, FBXO33, FBXO38, FBXO39, FBXO44, FBXO45, FBXO48, FBXW4, FBXW5, FBXW7, FBXW8, FBXW11, FEM1A, FEM1AL, FEM1B, FEM1C, FHIT, FOXF2, FOXRED2, FZR1, GABARAP, GBA, GCLC, GID4, GID8, GIPC1, GLMN, GNA12, GSK3A, GSK3B, HECTD1, HECTD3, HERC2, HERPUD1, HFE, HSP90B1, HSPA1A, HSPA1B, HSPA5, HSPBP1, IL33, ITCH, JKAMP, JKAMPL, KAT5, KBTBD6, KBTBD7, KCTD2, KCTD5, KCTD10, KCTD13, KCTD17, KIF14, KLHDC1, KLHDC2, KLHDC3, KLHDC10, KLHL15, KLHL20, KLHL22, KLHL40, KLHL42, LRRK2, LTN1, MAEA, MAN1A, MAN1B1, MAP1A, MAPK8, MAPK9, MARCHF6, MDM2, MTA1, MTM1, N4BP1, NAGLU, NCCRP1, NEDD4L, NEMF, NFE2L2, NHLRC1, NHLRC3, NKD2, NOP53, NPLOC4, NRROS, NSFL1C, NUB1, OGT, OS9, OTUD5, PABIR1, PABPN1L, PARK7, PBK, PCBP2, PCNP, PELI1, PHF20L1, PIAS1, PJA2, PLAA, PLK1, PLK2, PLK3, PML, PMP22, PPP2CB, PPP2R5C, PRICKLE1, PRKN, PSEN1, PSEN2, PSMA5, PSMB3, PSMB5, PSMC1, PSMC2, PSMC3, PSMC4, PSMC5, PSMC6, PSMD1, PSMD2, PSMD4, PSMD6, PSMD7, PSMD8, PSMD10, PSMD14, PSMF1, RACK1, RAD23A, RAD23B, RBCK1, RBX1, RCHY1, RFFL, RHOBTB3, RMND5A, RMND5B, RNF4, RNF5, RNF14, RNF19A, RNF19B, RNF34, RNF103, RNF121, RNF122, RNF126, RNF144A, RNF144B, RNF180, RNF185, RNF186, RNF187, RNF216, RNF217, RPL11, RYBP, SDCBP, SEC61B, SELENOS, SENP1, SGTA, SH3BGRL, SH3RF1, SH3RF2, SH3RF3, SHARPIN, SHH, SIAH1A, SIAH1B, SIAH2, SIAH3, SIRT1, SIRT2, SIRT6, SKP1, SKP2, SMAD7, SMARCC1, SMURF1, SMURF2, SOCS4, SOCS5, SPOP, SPOPFM1, SPOPFM2, SPOPFM3, SPOPL, SPSB1, SPSB2, SPSB3, SPSB4, STT3B, STUB1, STYX, SUMO1, SUMO2, SVIP, SYVN1, TAF9, TBL1X, TBL1XR1, TBX21, TDPOZ1, TDPOZ2, TDPOZ3, TDPOZ4, TDPOZ5, TDPOZ6, TDPOZ8, TDPOZ9, TLK2, TMEM67, TMEM129, TMUB1, TMUB2, TNFAIP1, TOPORS, TRIB1, TRIB2, TRIB3, TRIM2, TRIM3, TRIM9, TRIM13, TRIM25, TRIM38, TRIM39, TRIM63, TRIM71, TRIM72, TRIP4, TRIP12, TRPC4AP, TTC36, UBE2A, UBE2B, UBE2C, UBE2CBP, UBE2D3, UBE2G1, UBE2G2, UBE2H, UBE2J1, UBE2J2, UBE2K, UBE2S, UBE2U, UBE2V2, UBE2W, UBE4A, UBE4B, UBQLN1, UBQLN2, UBQLN4, UBR1, UBR2, UBR3, UBXN1, UBXN2A, UBXN2B, UBXN4, UBXN7, UBXN8, UBXN10, UBXN11, UCHL5, UFD1, UFL1, UMOD, USP5, USP7, USP9X, USP14, USP19, USP44, VCP, WAC, WDR26, WFS1, WNT10B, WWP1, WWP2, WWTR1, XPO1, YOD1, ZER1, ZFAND2A, ZFAND2B, ZFP598, ZNRF1, ZNRF2, ZSWIM8, ZYG11B, MTCO2P12, CTNS, FYN, MPV17L, DUOXA2, MT-CO2, STAT3, ZNF205, DUOXA1* |
