# Supplementary material for: A Model for the Development of Alzheimer’s Disease
Source: Genomics Proteomics Bioinformatics. 2025 Sep 23;23(6):qzaf087. doi: 10.1093/gpbjnl/qzaf087 (PMC13365266; doi:10.1093/gpbjnl/qzaf087)
Supplement: qzaf087_Supplementary_Data [file qzaf087_supplementary_data.zip › Table S3.docx]

**Table S3 GLS-associated reaction, UCP transporters, and pH-related acid loading transporters**

| **Cellular state or biological activity** | **Marker genes** |
| --- | --- |
| GLS | *GLS* |
| GLS2 | *GLS2* |
| Acid loading transporter | *SLC4A3, ATP2B1, ATP2B2, ATP2B3, ATP2B4* |
| UCP | *SLC25A14, UCP1, UCP2, UCP3, SLC25A27* |
| UCP2 | *UCP2* |
| UCP3 | *UCP3* |
| UCP4 | *SLC25A27* |
| UCP5 | *SLC25A14* |
| Acid-producing reactions | *GPX5, GPX7, GPX8, GPX6, GPX3, GPX1, GPX2, CPS1, TPO, KCNH7, XDH, NOS1, NOS2, NOS3, ALDH3A2, ADPRM, NUDT14, SCLY, FECH, FADS2, MOCS2, NUDT9, TRPM2, NUDT5, ALDH4A1, TYR, TYRP1, ALDH9A1, CYP27A1, CYP2G1P, CYP2U1, CYP2S1, CYP4X1, CYP2A6, CTU1, CTU2, PON1, PON2, PTER, MMAB, CAT, CP, FTH1, FTMT, FXN, ALDH2, SETD2, GMPS, PRMT1, PRMT6, CARM1, PRMT2, PRMT3, PRMT8, ADSS2, ADSS1, CYP51A1, MSMO1, ETHE1, ENTPD2, CTPS1, CTPS2, EZH2, EZH1, PRMT5, PRMT9, TRMT1, ALDH7A1, CAD, DPH5, PRDM2, SETDB2, EHMT1, EHMT2, SUV39H2, SUV39H1, NTMT1, DOT1L, HMOX1, HMOX2, SETMAR, NSD3, NSD2, SMYD2, NSD1, CYP2E1, ENTPD8, ENTPD1, ENTPD3, PRDM7, PRDM9, SMYD3, ASH1L, SETD1B, SETD1A, KMT2E, KMT2B, KMT2C, KMT2D, KMT2A, SMYD1, MTO1, GTPBP3, TFB2M, TFB1M, DIMT1, UQCR11, UQCR10, UQCRQ, UQCRB, UQCRH, UQCRC2, UQCRC1, UQCRFS1, CYC1, MT-CYB, CYP19A1, FADS1, DEGS1, ALDH1A3, ALDH1A1, ALDH1A2, HLCS, LIPT1, TST, LDHD, CYP11A1, ALDH5A1, TMEM189, UGDH, ALDH8A1, SCD, SCD5, FADS6, ALAD, CYP11B2, LCMT2, CPOX, ALDH1B1, ALDH3B2, ALDH3A1, ALDH3B1, EPX, LPO, PXDNL, PXDN, PRDX2, PRDX1, NUDT2* |
