# Supplementary material for: A Model for the Development of Alzheimer’s Disease
Source: Genomics Proteomics Bioinformatics. 2025 Sep 23;23(6):qzaf087. doi: 10.1093/gpbjnl/qzaf087 (PMC13365266; doi:10.1093/gpbjnl/qzaf087)
Supplement: qzaf087_Supplementary_Data [file qzaf087_supplementary_data.zip › Table S4.docx]

**Table S4 In pseudotime, raw data of LFR and 7 Events**

| **Time** | **transporters** | **GLS** | **UCP** | **Tau formation** | **Intracellular**  **pH**  **elevation** | **Endosomal**  **pH** | **Lysosomal**  **pH** | **FR** |
| --- | --- | --- | --- | --- | --- | --- | --- | --- |
| 0 | −2.52985 | −2.75592 | 3.651442 | 0.738587 | −3.13939 | −2.3109 | −2.14531 | 0.492587 |
| 1 | −2.083 | −3.35617 | −0.58631 | 1.172487 | −3.53505 | −3.53251 | −3.31694 | 0.47557 |
| 2 | −3.27622 | −3.24276 | 0.317641 | 1.380832 | −3.20145 | −2.44109 | −1.70173 | 0.478203 |
| 3 | −1.47409 | −3.57643 | −3.68258 | −2.36253 | −3.40566 | −2.85561 | −2.94447 | 0.480715 |
| 4 | 0.937236 | −0.83595 | −3.4784 | −3.48171 | −3.28896 | 6.837551 | 5.578054 | 0.477567 |
| 5 | −1.78163 | −2.09389 | −3.68327 | −3.63621 | 0.21349 | 7.136199 | 7.908396 | 0.495951 |
| 6 | −0.30828 | −1.27492 | −3.61667 | −3.65896 | −2.49781 | 3.697311 | 3.313183 | 0.47672 |
| 7 | −3.53273 | −3.12783 | −2.77882 | −1.13139 | −1.94866 | 4.660233 | 3.640026 | 0.476474 |
| 8 | −3.59683 | −3.09004 | −3.24233 | 0.249737 | −1.00136 | 1.167114 | 0.99891 | 0.461903 |
| 9 | −3.56696 | −3.4881 | −1.62221 | −0.83239 | −3.68652 | 1.833065 | 0.235415 | 0.44285 |
| 10 | −2.8296 | −3.0435 | −3.66448 | 0.928801 | −3.48278 | −0.74584 | −1.3823 | 0.468523 |
| 11 | −1.32927 | −3.44893 | −1.72818 | −0.60293 | −1.27875 | −2.21871 | −1.16477 | 0.446076 |
| 12 | 0.369217 | −1.84822 | −2.19014 | −2.32241 | −3.20383 | −3.57943 | −3.0899 | 0.445088 |
| 13 | 5.833047 | 0.976847 | −3.68222 | −1.93505 | −3.68764 | −1.0761 | −2.76011 | 0.44977 |
| 14 | 1.850463 | 7.894697 | −3.6674 | −3.52765 | −2.77566 | 1.955931 | 0.948931 | 0.456933 |
| 15 | 1.303136 | 7.457788 | −3.68475 | −3.58303 | −3.32707 | −1.68731 | −1.92904 | 0.461452 |
| 16 | −3.42873 | 3.231 | −3.67423 | −3.05258 | −3.68659 | −3.17684 | −3.13487 | 0.46766 |
| 17 | −3.65856 | −2.29078 | −3.68469 | −1.35051 | −2.95728 | −3.65609 | −3.6882 | 0.491533 |
| 18 | −3.58291 | −3.48147 | −3.5121 | 4.682095 | −3.68625 | −3.4336 | −3.48968 | 0.478704 |
| 19 | −3.47387 | −3.16806 | −3.47521 | 2.788324 | −3.32574 | −3.26518 | −3.32651 | 0.536364 |
| 20 | −3.52953 | −2.63187 | −1.87498 | −0.82669 | −3.64756 | −2.92371 | −3.18438 | 0.558734 |
| 21 | −3.67337 | −2.96146 | −3.13805 | 0.374931 | −3.67992 | −2.40182 | −2.762 | 0.549071 |
| 22 | −3.01565 | −3.2881 | −3.68786 | −0.58143 | 0.881856 | −3.4311 | −3.42276 | 0.580184 |
| 23 | −2.06196 | −3.4724 | −3.48736 | 1.444497 | 1.131571 | −3.53072 | −3.46068 | 0.569264 |
| 24 | −1.46612 | −3.61977 | −0.13667 | 8.056373 | 1.578256 | −3.00801 | −3.21734 | 0.564374 |
| 25 | −3.62027 | −0.36223 | −3.14763 | −1.71841 | −3.49252 | −3.68804 | −2.57817 | 0.54806 |
| 26 | −3.29108 | −1.22546 | −3.68765 | −2.16113 | −2.95288 | −3.12155 | −1.41061 | 0.546173 |
| 27 | −3.59459 | 3.49793 | −3.61842 | −3.26897 | −2.83822 | −3.68798 | −2.61288 | 0.510455 |
| 28 | −2.5619 | 2.815903 | −1.18794 | −3.51305 | −3.24079 | −3.68732 | −3.35637 | 0.485974 |
| 29 | −2.14242 | 5.402702 | −1.26319 | −3.34908 | −3.36757 | −3.61863 | −2.85071 | 0.500351 |
