# Supplementary material for: A Model for the Development of Alzheimer’s Disease
Source: Genomics Proteomics Bioinformatics. 2025 Sep 23;23(6):qzaf087. doi: 10.1093/gpbjnl/qzaf087 (PMC13365266; doi:10.1093/gpbjnl/qzaf087)
Supplement: qzaf087_Supplementary_Data [file qzaf087_supplementary_data.zip › Table S5.docx]

**Table S5 In pseudotime, raw data of Neuron death and 4 Events**

| **Time** | **Amyloid beta**  **formation** | **Extracellular acidity** | **Intracellular pH elevation** | **Tau formation** | **Neuron death** |
| --- | --- | --- | --- | --- | --- |
| 0 | 32.25612 | 47.25035 | −4.5068 | −1.73923 | 0.574631 |
| 1 | 25.07704 | 34.79839 | 0.618244 | 3.791489 | 0.570222 |
| 2 | 28.56704 | 42.20411 | 1.288234 | 1.896825 | 0.573785 |
| 3 | 27.63045 | 38.69986 | 2.481605 | 2.575197 | 0.562698 |
| 4 | 26.25769 | 36.36632 | −3.01676 | 0.048811 | 0.569787 |
| 5 | 25.9389 | 36.62798 | −5.51598 | −2.50745 | 0.572925 |
| 6 | 27.15263 | 39.54422 | −6.69498 | −3.29083 | 0.563678 |
| 7 | 26.98432 | 38.80317 | −5.35121 | −1.8085 | 0.574324 |
| 8 | 31.49833 | 47.40027 | −6.02758 | −2.41538 | 0.566046 |
| 9 | 44.37646 | 68.37781 | −3.31536 | −1.17266 | 0.569673 |
| 10 | 41.58606 | 61.87346 | 4.359723 | 4.234152 | 0.573034 |
| 11 | 37.73053 | 60.81388 | 0.563841 | −0.58309 | 0.574699 |
| 12 | 41.34228 | 66.13155 | 7.082083 | 3.715401 | 0.571492 |
| 13 | 35.46413 | 54.75861 | 1.641953 | 1.025303 | 0.566264 |
| 14 | 33.70445 | 61.26605 | −7.03098 | −3.67452 | 0.567966 |
| 15 | 45.4188 | 77.56797 | −6.85499 | −3.57777 | 0.560669 |
| 16 | 52.3645 | 79.26765 | −7.22919 | −3.68223 | 0.570683 |
| 17 | 53.53414 | 86.93437 | −7.27121 | −3.51373 | 0.573923 |
| 18 | 77.71972 | 108.7708 | −1.13369 | −1.19827 | 0.575348 |
| 19 | 68.34562 | 92.75721 | −2.84386 | −1.91199 | 0.575529 |
| 20 | 59.72709 | 83.4756 | −3.42669 | −1.14526 | 0.574403 |
| 21 | 54.7833 | 75.44546 | −0.70938 | 1.586315 | 0.56632 |
| 22 | 49.96451 | 68.22181 | −5.69663 | −2.02769 | 0.574289 |
| 23 | 33.63458 | 44.43044 | −4.84613 | −1.21266 | 0.591765 |
| 24 | 31.0213 | 39.24799 | −4.50791 | −0.8082 | 0.602014 |
| 25 | 36.52504 | 43.71549 | −5.89691 | −2.4145 | 0.620698 |
| 26 | 41.20984 | 48.44101 | −5.59781 | −2.16537 | 0.623211 |
| 27 | 58.25386 | 63.96841 | −0.156 | 1.764721 | 0.614837 |
| 28 | 51.24422 | 65.24827 | −7.53655 | −3.41276 | 0.612008 |
| 29 | 57.33791 | 70.58372 | −6.04223 | −3.65284 | 0.602241 |
