# Supplementary material for: A Model for the Development of Alzheimer’s Disease
Source: Genomics Proteomics Bioinformatics. 2025 Sep 23;23(6):qzaf087. doi: 10.1093/gpbjnl/qzaf087 (PMC13365266; doi:10.1093/gpbjnl/qzaf087)
Supplement: qzaf087_Supplementary_Data [file qzaf087_supplementary_data.zip › Table S6.docx]

**Table S6 Lactate-producing enzymes, and monocarboxylate transporters**

| **Group** | **Transcript** | **Gene** | **Case** | **Normal** | **Log_2_FC** | ***P* value** | ***P* adj** |
| --- | --- | --- | --- | --- | --- | --- | --- |
| MCI | ENST00000540430.5 | *LDHA* | 528.7361 | 705.6519 | −0.69565 | 0.018641 | 0.519074 |
| MCI | ENST00000542179.1 | *LDHA* | 235.7184 | 189.2034 | 0.301555 | 0.090669 | 0.856995 |
| MCI | ENST00000437325.2 | *LDHA* | 3.183453 | 5.191039 | −0.29196 | 0.108776 | 0.891627 |
| MCI | MSTRG.11016.22 | *LDHA* | 44.46299 | 116.2351 | −5.50431 | 0.1551 | 0.960264 |
| MCI | ENST00000538451.1 | *LDHA* | 183.2573 | 215.9434 | −0.23483 | 0.196997 | 0.995543 |
| MCI | ENST00000394996.2 | *LDHA* | 1.016107 | 1.245959 | −0.24304 | 0.329721 | 1 |
| MCI | ENST00000430553.6 | *LDHA* | 1121.837 | 1312.198 | −0.2179 | 0.333767 | 1 |
| MCI | MSTRG.11016.5 | *LDHA* | 86.9179 | 59.82111 | 1.222609 | 0.345234 | 1 |
| MCI | ENST00000652129.1 | *LDHA* | 27.83675 | 36.26566 | −0.37192 | 0.375408 | 1 |
| MCI | ENST00000398295.3 | *LDHA* | 1.307127 | 1.269087 | 0.234389 | 0.405643 | 1 |
| MCI | ENST00000375710.7 | *LDHA* | 458.1318 | 425.4083 | 0.134313 | 0.495529 | 1 |
| MCI | ENST00000396222.6 | *LDHA* | 221.2177 | 237.2965 | −0.11357 | 0.522281 | 1 |
| MCI | MSTRG.11016.1 | *LDHA* | 706.1548 | 806.4026 | −0.17487 | 0.537503 | 1 |
| MCI | MSTRG.11016.20 | *LDHA* | 134.2459 | 186.0702 | −0.55697 | 0.621259 | 1 |
| MCI | MSTRG.11016.21 | *LDHA* | 23.79716 | 12.55301 | 0.168875 | 0.65906 | 1 |
| MCI | ENST00000227157.8 | *LDHA* | 406.6567 | 447.3849 | −0.06278 | 0.70391 | 1 |
| MCI | MSTRG.11016.10 | *LDHA* | 213.5268 | 186.0065 | 0.248115 | 0.743594 | 1 |
| MCI | MSTRG.11016.3 | *LDHA* | 206.7112 | 155.4987 | 0.389588 | 0.781365 | 1 |
| MCI | ENST00000379412.9 | *LDHA* | 614.1159 | 606.8247 | 0.088371 | 0.784113 | 1 |
| MCI | ENST00000397561.3 | *LDHA* | 41.95989 | 43.15236 | −0.03481 | 0.819262 | 1 |
| MCI | ENST00000448312.2 | *LDHA* | 2.420392 | 2.447554 | 0.044472 | 0.84927 | 1 |
| MCI | ENST00000545215.5 | *LDHA* | 1831.598 | 1803.468 | 0.019154 | 0.904292 | 1 |
| MCI | MSTRG.11016.19 | *LDHA* | 96.50774 | 90.80989 | 0.010833 | 0.924105 | 1 |
| MCI | ENST00000509421.1 | *LDHA* | 0.042499 | 0.04287 | −0.05469 | 1 | 1 |
| MCI | MSTRG.14687.8 | *LDHB* | 2035.1 | 1936.201 | −0.37233 | 0.423511 | 1 |
| MCI | ENST00000470280.1 | *LDHB* | 5.862688 | 6.890425 | −0.09705 | 0.465156 | 1 |
| MCI | MSTRG.14687.5 | *LDHB* | 1365.175 | 1539.144 | −0.18749 | 0.500994 | 1 |
| MCI | ENST00000456969.2 | *LDHB* | 0.362268 | 0.421488 | −0.20435 | 0.555963 | 1 |
| MCI | ENST00000396076.5 | *LDHB* | 5243.979 | 4943.141 | 0.066013 | 0.664128 | 1 |
| MCI | MSTRG.14687.7 | *LDHB* | 148.0779 | 193.0706 | 0.281515 | 0.860305 | 1 |
| MCI | ENST00000455730.2 | *LDHB* | 3.224953 | 3.247989 | 0.031642 | 0.869478 | 1 |
| MCI | ENST00000673047.2 | *LDHB* | 11583.79 | 11725.05 | 0.01543 | 0.885474 | 1 |
| MCI | ENST00000280704.8 | *LDHC* | 2.393039 | 3.066313 | −0.26873 | 0.440665 | 1 |
| MCI | ENST00000396215.7 | *LDHC* | 1.811266 | 2.51806 | −0.32994 | 0.738922 | 1 |
| MCI | ENST00000536880.5 | *LDHC* | 0.044357 | 0.149268 | −0.35638 | 0.822293 | 1 |
| MCI | ENST00000544105.5 | *LDHC* | 0.181588 | 0.124802 | 0.191833 | 0.823899 | 1 |
| MCI | ENST00000545848.5 | *LDHC* | 0.081963 | 0.102454 | −0.12895 | 0.929383 | 1 |
| MCI | ENST00000535809.1 | *LDHC* | 0.15278 | 0.17849 | −0.04732 | 1 | 1 |
| MCI | ENST00000546146.5 | *LDHC* | 0.03646 | 0.030059 | −0.02911 | 1 | 1 |
| AD | MSTRG.11016.19 | *LDHA* | 104.178 | 89.29306 | 0.201387 | 0.055143 | 0.428797 |
| AD | ENST00000430553.6 | *LDHA* | 988.8649 | 1289.119 | −0.37733 | 0.066905 | 0.466338 |
| AD | ENST00000437325.2 | *LDHA* | 3.716113 | 5.091308 | −0.28145 | 0.090114 | 0.5282 |
| AD | MSTRG.11016.22 | *LDHA* | 55.89878 | 114.3718 | −6.35139 | 0.12216 | 0.594426 |
| AD | ENST00000545215.5 | *LDHA* | 1551.71 | 1772.41 | −0.19665 | 0.152061 | 0.644508 |
| AD | ENST00000398295.3 | *LDHA* | 1.464354 | 1.245894 | 0.329773 | 0.161878 | 0.658292 |
| AD | ENST00000397561.3 | *LDHA* | 37.51298 | 42.39355 | −0.17038 | 0.199042 | 0.703768 |
| AD | ENST00000394996.2 | *LDHA* | 0.973928 | 1.22413 | −0.27587 | 0.23124 | 0.739721 |
| AD | ENST00000652129.1 | *LDHA* | 27.19808 | 35.57989 | −0.4159 | 0.289351 | 0.789783 |
| AD | ENST00000538451.1 | *LDHA* | 188.5394 | 212.3921 | −0.17964 | 0.292358 | 0.792178 |
| AD | MSTRG.11016.5 | *LDHA* | 75.03673 | 58.53183 | −1.58409 | 0.295071 | 0.794245 |
| AD | ENST00000227157.8 | *LDHA* | 400.2343 | 439.4687 | −0.12658 | 0.419696 | 0.870282 |
| AD | ENST00000540430.5 | *LDHA* | 642.7322 | 692.3941 | −0.13243 | 0.624658 | 0.940775 |
| AD | ENST00000379412.9 | *LDHA* | 518.3714 | 596.2904 | −0.1385 | 0.629187 | 0.942024 |
| AD | ENST00000448312.2 | *LDHA* | 2.480952 | 2.407671 | 0.086332 | 0.684565 | 0.954433 |
| AD | ENST00000396222.6 | *LDHA* | 225.2769 | 233.2123 | −0.06422 | 0.710801 | 0.959536 |
| AD | MSTRG.11016.3 | *LDHA* | 178.7161 | 152.7705 | 0.445655 | 0.71223 | 0.959926 |
| AD | MSTRG.11016.10 | *LDHA* | 205.8669 | 182.7755 | −0.17524 | 0.807584 | 0.976857 |
| AD | MSTRG.11016.20 | *LDHA* | 154.7399 | 182.4589 | −0.39775 | 0.822455 | 0.979 |
| AD | ENST00000542179.1 | *LDHA* | 192.1217 | 186.0902 | 0.0304 | 0.850029 | 0.983015 |
| AD | ENST00000375710.7 | *LDHA* | 415.6014 | 418.3174 | 0.022845 | 0.902307 | 0.990251 |
| AD | ENST00000509421.1 | *LDHA* | 0.060816 | 0.041959 | 0.12 | 0.925514 | 1 |
| AD | MSTRG.11016.1 | *LDHA* | 779.9035 | 791.3548 | −0.02003 | 0.937158 | 0.994189 |
| AD | MSTRG.11016.21 | *LDHA* | 0.163187 | 12.31229 | −0.12985 | 1 | 1 |
| AD | ENST00000455730.2 | *LDHB* | 4.069332 | 3.19864 | 0.392391 | 0.023272 | 0.287274 |
| AD | ENST00000470280.1 | *LDHB* | 7.681787 | 6.765809 | 0.26502 | 0.042037 | 0.380219 |
| AD | ENST00000396076.5 | *LDHB* | 4337.53 | 4857.828 | −0.18572 | 0.185224 | 0.687379 |
| AD | MSTRG.14687.5 | *LDHB* | 1431.777 | 1511.425 | −0.11682 | 0.670705 | 0.951623 |
| AD | MSTRG.14687.8 | *LDHB* | 2191.781 | 1903.962 | −0.17201 | 0.691232 | 0.95578 |
| AD | ENST00000456969.2 | *LDHB* | 0.359134 | 0.414054 | −0.11497 | 0.717033 | 1 |
| AD | ENST00000673047.2 | *LDHB* | 11052.27 | 11521.62 | 0.014893 | 0.872415 | 0.985986 |
| AD | MSTRG.14687.7 | *LDHB* | 59.71575 | 189.9493 | −0.21878 | 0.887563 | 0.988382 |
| AD | ENST00000455730.2 | *LDHB* | 4.069332 | 3.19864 | 0.392391 | 0.023272 | 0.287274 |
| AD | ENST00000280704.8 | *LDHC* | 2.111653 | 3.015 | −0.4601 | 0.133472 | 0.61458 |
| AD | ENST00000396215.7 | *LDHC* | 1.055423 | 2.473059 | −1.02446 | 0.285844 | 0.787049 |
| AD | ENST00000545848.5 | *LDHC* | 0.296123 | 0.100231 | 0.554991 | 0.586902 | 1 |
| AD | ENST00000544105.5 | *LDHC* | 0.219342 | 0.122876 | 0.362949 | 0.726496 | 1 |
| AD | ENST00000535809.1 | *LDHC* | 0.067363 | 0.174638 | −0.32614 | 0.800563 | 1 |
| AD | ENST00000536880.5 | *LDHC* | 0.06856 | 0.146573 | −0.15227 | 0.881327 | 1 |
