# Supplementary material for: A Model for the Development of Alzheimer’s Disease
Source: Genomics Proteomics Bioinformatics. 2025 Sep 23;23(6):qzaf087. doi: 10.1093/gpbjnl/qzaf087 (PMC13365266; doi:10.1093/gpbjnl/qzaf087)
Supplement: qzaf087_Supplementary_Data [file qzaf087_supplementary_data.zip › Table S7.docx]

**Table S7 The remaining pertinent genes**

| **Cellular state or biological activity** | **Marker genes** |
| --- | --- |
| Neuron death in response to hydrogen peroxide | *SYMBOL, FBXO7, IL10,*  *NR4A3, PARK7, PINK1, RACK1, REG3B* |
| Neuron death in response to oxidative stress | *SYMBOL, APP, ARL6IP5, ATF4, ATG7, CTNNB1, FBXO7, FBXW7, FZD1, GFER, HIF1A, IL10, ITGAM, LANCL1, MCL1, MEAK7, NCOA7, NONO, NR4A3, OXR1, PARK7, PARP1, PINK1, PRKN, RACK1, REG3B, REST, SLC1A1, SLC7A11, TBC1D24, TLDC2, TLR4, TLR6, TNF, TREM2, TRPM2, TSC1, WNT1* |
| Hydrogen peroxide biosynthetic process | *MTCO2P12, CTNS, FYN, MPV17L, DUOXA2, MT-CO2, STAT3, ZNF205, DUOXA1* |
| Positive regulation of neuron apoptotic process | *ABL1, AGER, AGRN, AIFM1, APP, ASCL1, ATF2, ATF4, ATM, BACE1, BAG1, BAX, BBC3, BCL2L11, CASP1, CASP2, CASP3, CASP6, CASP9, CCL3, CDC34, CDC34B, CDC42, CDK5, CDK5R1, CTNNB1, CTSZ, DDIT3, EGR1, EPHA7, FAS, FASL, FBXW7, FGFR3, FIS1, GRIK2, GRIK5, GRN, GSK3A, GSK3B, HDAC3, HDAC4, HRK, IL1B, IL18, ITGA1, ITGAM, JUN, KCNMA1, LCN2, MAP2K4, MAP2K7, MAP3K11, MAPK8, MCL1, MUSK, MYB, MYBL2, NCF2, NF1, NFATC4, NQO1, NQO2, NR3C1, NUPR1, OPTN, PAK3, PAWR, PCSK9, PIN1, PIN1RT1, PITX3, PMAIP1, POU4F1, PPP2R2B, PRNP, PTPRF, RAPSN, RHOA, SRPK2, TFAP2A, TFAP2B, TGFB2, TNF, TNFRSF1A, TRAF7, TRP53, TYROBP, UBE2M* |
| Na-K-ATPase | *ATP1B4, ATP1B2, ATP1B3, ATP1B1, ATP1A4, ATP1A3, ATP1A2, ATP1A1* |
| Hyponatremia | *HMBS, NR3C2, AVPR2, SCNN1G, CA12, SLC26A3, CPOX, SCN4A, OCRL, BSND, IKBKAP, PRF1, SCNN1A, SARS2, CLCNKA, CYP11B2, CLCNKB, CTNS, NR0B1, SCNN1B* |
| Chronic inflammatory response | *CEBPB, ADORA2B, CYP19A1, AHCYL1, CX3CR1, FOXP3, GJA1, CAMP, LTA, TNFAIP3, VNN1, IL10, IL4, IDO1, S100A9, TNF, AHCY, UNC13D, PDE5A* |
| Inflammatory cell apoptotic process | *CCL5, CCR5, MEF2C, SELENOS, FAS, PIK3CB, ST6GAL1, PIK3CD, CXCR2, ITPKB, NOD2, ZMPSTE24, ANXA1, SIRT1, CDKN2A, IFNG, CD44, FCGR2B, FASLG, PLEKHO2, GHSR, IL18, SLC7A11, IL6, PTEN, TCP1, ITGAM* |
| Synaptic vesicle recycling | *BCL2L1, ACTG1, FCHO2, SNAP91, STX1B, NOSTRIN, CANX, ITSN1, ITSN2, NAPB, SYNJ1, SCAMP5, RAB5A, SYT8, RAB27B, SYT1, SYT2, SLC2A4, SYT5, SYT7, SYP, ACTB, DNM1, DNM3, DNM2, AP2B1, LRRK2, SYT11, PCLO, TBC1D24, GRN, AMPH, ABCA13, DNAJC6, SNCG, SNCB, STON2, STON1, SNCA, BTBD8, STX1A, 4932438A13RIK, AP3M2, SH3GL1, PLAA, SH3GL3, SH3GL2, ARF6, AP2M1, AP3D1, FGF14, NLGN2, NLGN3, NLGN1, GRIPAP1, VAMP4, PIP5K1C, SLC17A7, CDK5, CYFIP1, CLTC, AP3S2, AP3S1, AP1S2, BIN1, AP3B1, AP3B2, DGKQ, OPHN1, TOR1A, SYNJ2, BTBD9, PACSIN1, GIT1, GIT2, RAB3A, DENND1A, NLGN4L, VAMP2, CD24A, ROCK1, SCRIB, DNM1L, PPP3CB, PPP3CC, PICALM* |
| Amyloid beta formation | *ABCA2, ABCA7, ABCG1, ADAM10, APH1A, APH1B, nan, APOE, BACE1, BIN1, CASP3, CHRNA7, CLU, DYRK1A, EFNA1, EPHA4, GGA3, GSAP, GSK3A, HAP1, IFNG, IFNGR1, IGF1, LRRTM3, NCSTN, NTRK2, OLFM1, PICALM, PIN1, PRNP, PSEN1, PSEN2, PSENEN, RELA, ROCK1, ROCK2, RTN1, RTN2, RTN3, RTN4, SLC2A13, SORL1, SP1, SPON1, TMED10, TNF* |
| Amyloid precursor protein catabolic process | *ABCA2, ABCA7, ABCG1, ADAM9, ADAM10, ADAM17, ADAM19, AGER, APH1A, APH1B, APOE, APP, BACE1, BIN1, CASP3, CHRNA7, CLN3, CLU, DHCR24, DYRK1A, EFNA1, EFNA3, EPHA4, FLOT2, GGA3, GSAP, GSK3A, HAP1, IFNG, IFNGR1, IGF1, LRRTM3, LYN, NCSTN, NTRK2, OLFM1, PICALM, PIN1, nan, PRNP, PSEN1, PSEN2, PSENEN, RANBP9, RELA, ROCK1, ROCK2, RTN1, RTN2, RTN3, RTN4, SLC2A13, SORL1, SP1, SPON1, TMED10, TNF, UNC13A* |
| Intracellular pH elevation | *NOX1, SLC26A3, CLN3, CFTR, SLC26A6* |
| Intracellular pH reduction | *AVPR1A, RAB39A, CA7, RAB7A, RAB38, TCIRG1, SLC9A7, SLC11A1, PPT1, UBE3A, ATP6AP2, SLC12A5, CLN6, CLN5, CLN3, ATP6V0D2, ATP6V0D1, AQP11, SNAPIN, ATP6V0C, CLIC4, TMEM106B, TMEM9, FASLG, RAB20, GRN, GPR89A, RNASEK, DMXL2, DMXL1, CA2, CCDC115, AVP, TTPA, ATP6V0A4, ATP6V0A1, ATP6V0A2, SLAMF8, TMEM199, BCL2* |
| Mitochondrial pH | *UCP2, UCP3, UCP4, SLC25A14, LDHA, LDHB* |
| Endosomal lumen acidification | *AQP11, FASLG, RNASEK, TMEM9* |
| Lysosomal lumen acidification | *ATP6AP2, ATP6V0C, CCDC115, CLN3, CLN5, CLN6, GRN, PPT1, SNAPIN, TMEM9, TMEM106B, TMEM199* |
| Lysosomal lumen pH elevation | *CLN3* |
| Aquaporins | *AQP1, AQP10, AQP11, AQP12A, AQP12B, AQP1AQP11, AQP2, AQP3, AQP4, AQP5, AQP6, AQP6AQP6, AQP7, AQP8, AQP9, MIP* |
| Excitatory postsynaptic potential | *ADORA1, ADORA2A, ADRB2, APP, ATXN1, BAIAP2, BDNF, BEGAIN, CACNB3, CDK5, CELF4, CHRNA3, CHRNA4, CHRNA7, CKAP5, CNTNAP2, CUX2, DBN1, DGKI, DLG4, DMPK, DRD4, DVL1, EIF4A3, EIF4A3L1, EIF4A3L2, GHRL, GRID2, GRIK1, GRIK2, GRIK5, GRIN1, GRIN2A, GRIN2B, GRIN2C, GRIN2D, GRIP2, GRK2, GSK3B, HCRT, IGSF11, LRRK2, MAPK8IP2, MECP2, MEF2C, MET, MPP2, MTMR2, NETO1, NETO2, NGFR, NLGN1, NLGN2, NLGN3, NPAS4, NPFF, NPY2R, NRXN1, OPRM1, P2RX4, P2RX5, PCLO, PLK2, PPP1R9A, PPP3CA, PRKAR1B, PRKCZ, PRKN, PTEN, PTK2B, RAB3GAP1, RELN, RGS4, RIMS1, RIMS2, S1PRCBLN1, SEZ6, SH3GL1, SHANK1, SHANK3, SLC17A7, SLC29A1, SLC8A2, SLC8A3, SNCA, SSH1, STX1A, STX1B, TBC1D24, TMEM108, TMEM25, WNT7AFDN, ZMYND8* |
| Copper ion binding | *ADNP, AHCY, ANG F5, ANG2, ANG3, ANG4, ANG5, ANG6, AOC1, AOC1L1, AOC1L2, AOC1L3, AOC2, AOC3, APOA4, ATOX1, ATP7A, ATP7B, CCS, COA6, COMMD1, COX11, COX17, CP, CUTA, CUTAL, CUTC, DBH, F8, GPC1, HAMP, HEPH, HEPHL1, IL1A, LACC1, LOX, LOXL1, LOXL2, LOXL3, LOXL4, MOXD1, MOXD2, MT-CO2, MT1, MT3, MUC2, OR4E2, OR5AR1, P2RX2, P2RX4, P2RXA, PAM, PARK7, PRN, PRND, PRNP, RNPEP, S100A13, S100A5, SCO2, SLC11A2, SNCA, SNCB, SNCG, SOD1, SOD3, SUMF1, TRP53, TYR. ​* |
| Stress response to copper ion | *MT2 MUC2 PARK7 MT1 ATP7A* |
| Extracellular copper | *ACPP, ANG, ANGPT1, ANGPT2, ANGPTL1, ANGPTL2, ANGPTL4, AOC1, CDCPSLC11A2, CP, CPA1, CPA2, CPB2, CPN2, CPQ, DBH, F8, FGF8, GPC1, LOX, LOXL1, LOXL2, LOXL3, PAM, S100A13, SNCA, SOD1, SOD3. ​* |
| Proteasome | *PSMD9 PSMD8 PSMB10 PSMD5 PSMD4 PSMD7 PSMD6 PSMD1 PSMD3 PSMD2 PSMF1 PSMA2 PSMA3 PSMA1 PSMA6 PSMA7 PSMA4 PSMA5 PSMC1 PSMA8 PSMC3 PSMC4 PSMC5 PSMC6 PSME4 ADRM1 PSMEPSME3 PSME1 PSMC2 SEM1 PSMD14 PSMD11 PSMD10 PSMD13 PSMD12 PSMB9 PSMB8 PSMB7 PSMB6 PSMB5 PSMB4 PSMB3 PSMB2 PSMB1* |
| Chloride channels | *CLCN3 CLCN2 CLCN7 CLCN6 CLCN5 CLCN4 CLCNKA* |
| VDAC | *VDAC3 VDAC2 VDAC1* |
| Synapse pruning | *ADGRB3, C1QA, C1QB, C1QC, C1QL1, C3, CDK5, CX3CR1, EPHA4, ITGAM, ITGB1, NGEF, PLXNC1, TREM2. ​​* |
