# Supplementary material for: A Model for the Development of Alzheimer’s Disease
Source: Genomics Proteomics Bioinformatics. 2025 Sep 23;23(6):qzaf087. doi: 10.1093/gpbjnl/qzaf087 (PMC13365266; doi:10.1093/gpbjnl/qzaf087)
Supplement: qzaf087_Supplementary_Data [file qzaf087_supplementary_data.zip › Table S8.docx]

**Table S8 All software used in this article**

| **Resource** | **Source** | **Version** |
| --- | --- | --- |
| **Database** | | |
| ROSMAP RNA-seq | [(Bennett et. Al, 2012)](https://www.ncbi.nlm.nih.gov/pmc/articles/PMC3409291/) | syn3219045 |
| ROSMAP snRNA-seq | [(Yingyue Zhou et al., 2020)](https://www.nature.com/articles/s41591-019-0695-9#Sec45) | syn21670836 |
| MSBB RNA-seq | [(Minghui Wang et.al., 2018)](https://www.nature.com/articles/sdata2018185) | syn3159438 |
| Gene Expression Omnibus | [(Brad A Friedman et al., 2018)](https://www.cell.com/cell-reports/fulltext/S2211-1247(17)31903-4?_returnURL=https%3A%2F%2Flinkinghub.elsevier.com%2Fretrieve%2Fpii%2FS2211124717319034%3Fshowall%3Dtrue) | GSE95587 |
| **Software and algorithms** | | |
| RStudio | <https://www.rstudio.com/> | v1.4 |
| R | <https://www.r-project.org/> | v4.1.2 |
| Anaconda Python | [https://www.anaconda.com](https://www.anaconda.com/) | v3.8.0 |
| Anaconda Python | [https://www.anaconda.com](https://www.anaconda.com/) | v2.7.0 |
| SOAPnuke | <https://github.com/BGI-flexlab/SOAPnuke> | v2.x |
| HISAT2 | <http://daehwankimlab.github.io/hisat2/> | v2.2.1 |
| samtools | <http://www.htslib.org/> | v1.9.0 |
| StringTie | <https://ccb.jhu.edu/software/stringtie/> | v2.1.4 |
| ballgown | [https://www.bioconductor.org](https://www.bioconductor.org/packages/release/bioc/html/ballgown.html) | v2.28.0 |
| gffcompare | [https://ccb.jhu.edu/software/stringtie](https://ccb.jhu.edu/software/stringtie/gffcompare.shtml) | v0.11.7 |
| eggNOG-mapper | <http://eggnog-mapper.embl.de/> | v2 |
| CNCI | <http://cnit.noncode.org/CNIT/download> | v1.0 |
| CPAT | <http://rna-cpat.sourceforge.net/> | v2.0.0 |
| CPC2 | <http://cpc2.gao-lab.org/> | v0.1 |
| CPPred | <http://www.rnabinding.com/CPPred/> | v1 |
| PLEK | <http://202.200.112.245/plek/> | V1.2 |
| DESeq2 | <https://www.bioconductor.org/> | v1.36.0 |
| clusterProfiler | <https://www.bioconductor.org/> | v3.12.0 |
| PCA | [https://scikit-learn.org](https://scikit-learn.org/stable/modules/generated/sklearn.decomposition.PCA.html) | v.1.1.1 |
| OLS | <https://www.statsmodels.org/stable/api.html> | v0.13.2 |
| semopy | [https://semopy.com/#whatis](https://semopy.com/%23whatis) | v2.0 |
| Breaking Cycles In Noisy Hierarchies | <https://github.com/zhenv5/breaking_cycles_in_noisy_hierarchies> | v1 |
| networkx | <https://networkx.org/> | v2.8.5 |
| gsva | [https://www.bioconductor.org](https://www.bioconductor.org/packages/release/bioc/html/GSVA.html) | v1.44.2 |
| cellranger | [https://support.10xgenomics.com](https://support.10xgenomics.com/single-cell-gene-expression/software/pipelines/latest/what-is-cell-ranger) | v6.0.0 |
| Loupe | [https://www.10xgenomics.com](https://www.10xgenomics.com/products/loupe-browser/downloads) | v.6.1.0 |
