# Supplementary figures and images for: A Model for the Development of Alzheimer’s Disease
Source: Genomics Proteomics Bioinformatics. 2025 Sep 23;23(6):qzaf087. doi: 10.1093/gpbjnl/qzaf087 (PMC13365266; doi:10.1093/gpbjnl/qzaf087)

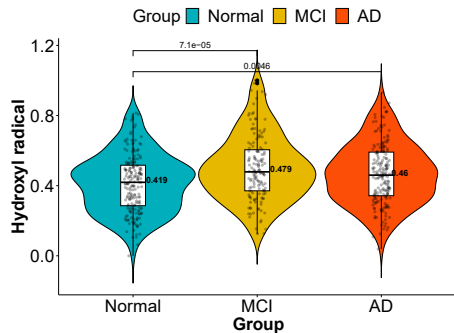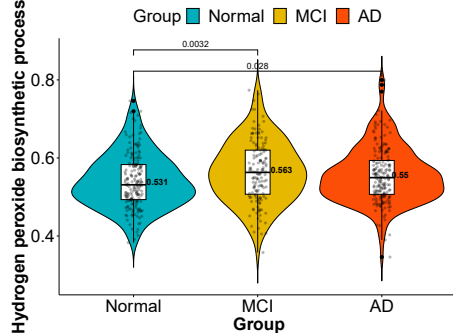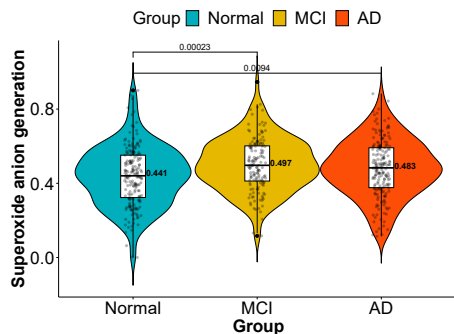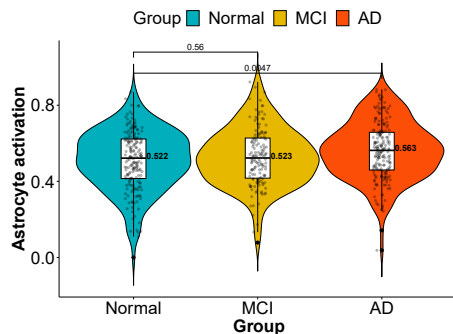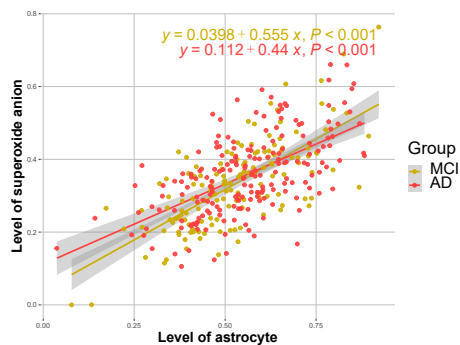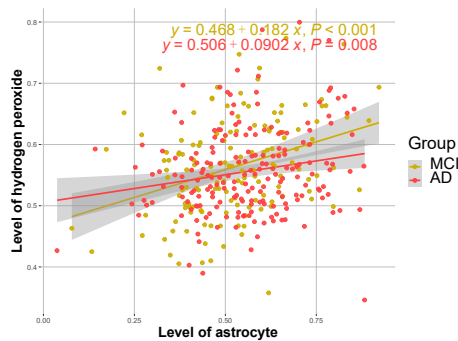

Supplement: qzaf087_Supplementary_Data [file qzaf087_supplementary_data.zip › Figure S1.pdf]

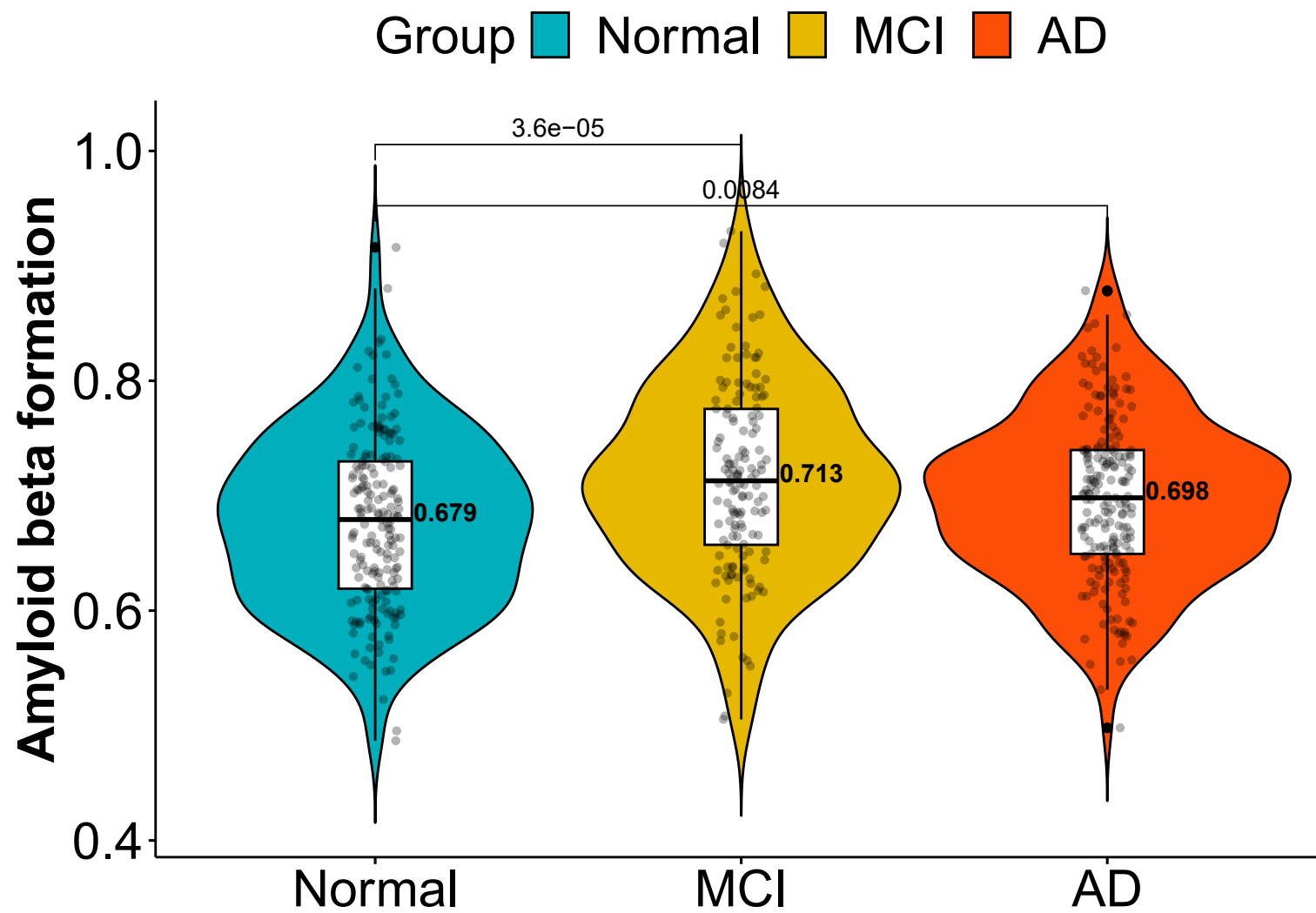

Supplement: qzaf087_Supplementary_Data [file qzaf087_supplementary_data.zip › Figure S10.pdf]

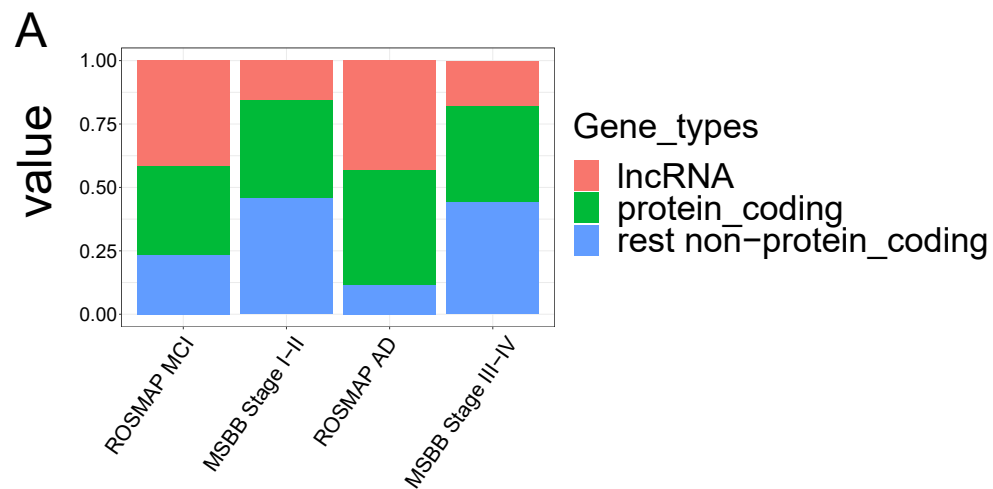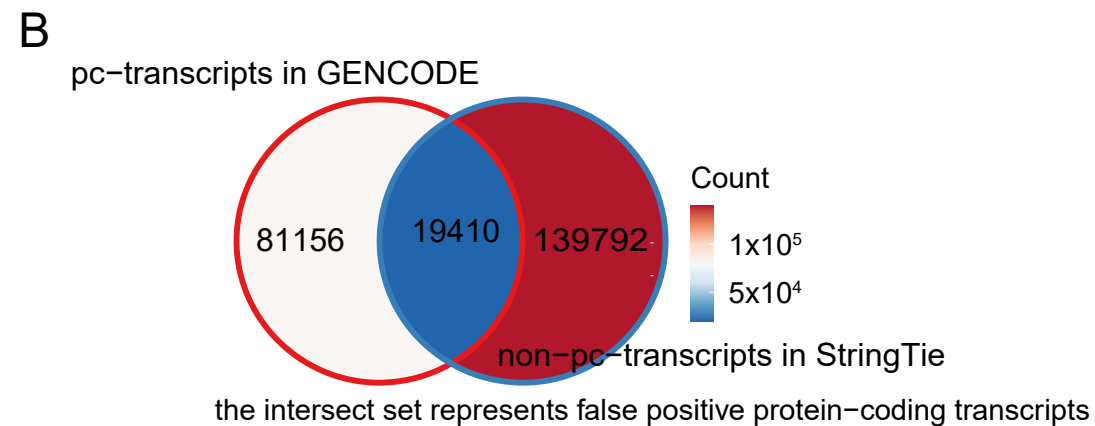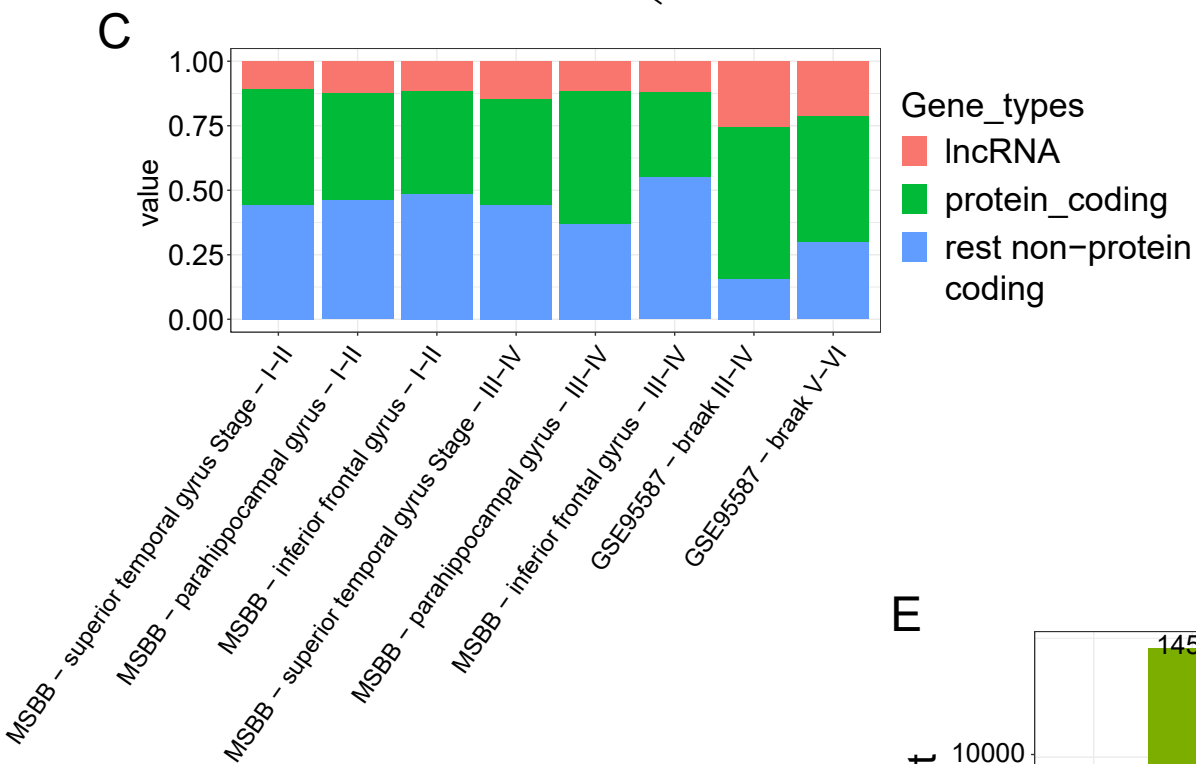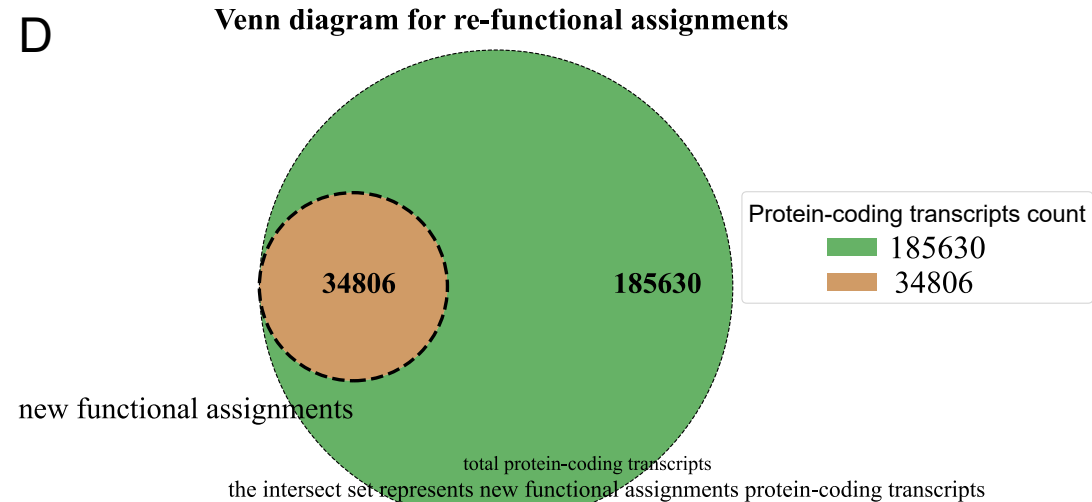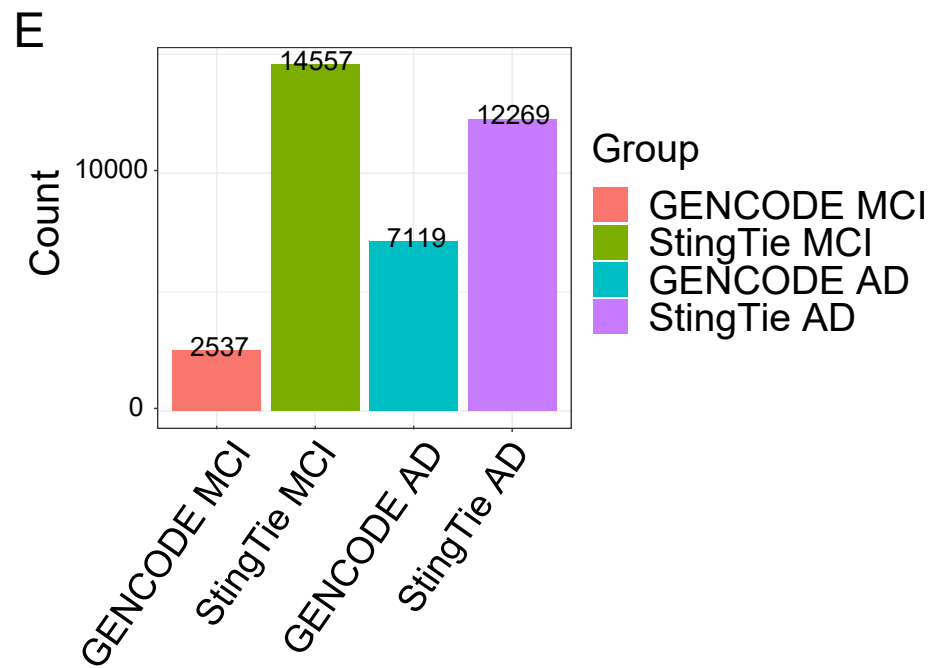

Supplement: qzaf087_Supplementary_Data [file qzaf087_supplementary_data.zip › Figure S12.pdf]

A

## Glutaminase related DETs total TPM

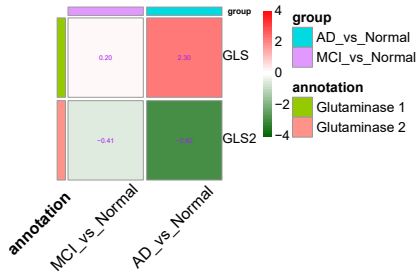

B

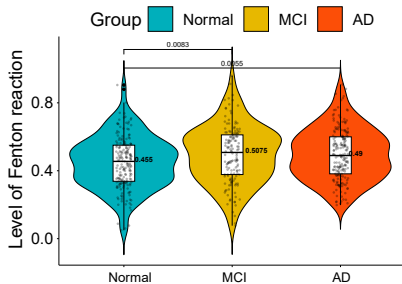

C

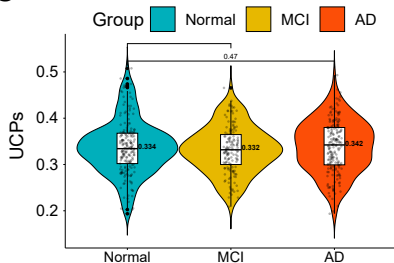

D

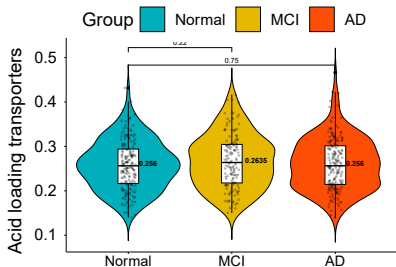

Supplement: qzaf087_Supplementary_Data [file qzaf087_supplementary_data.zip › Figure S2.pdf]

A

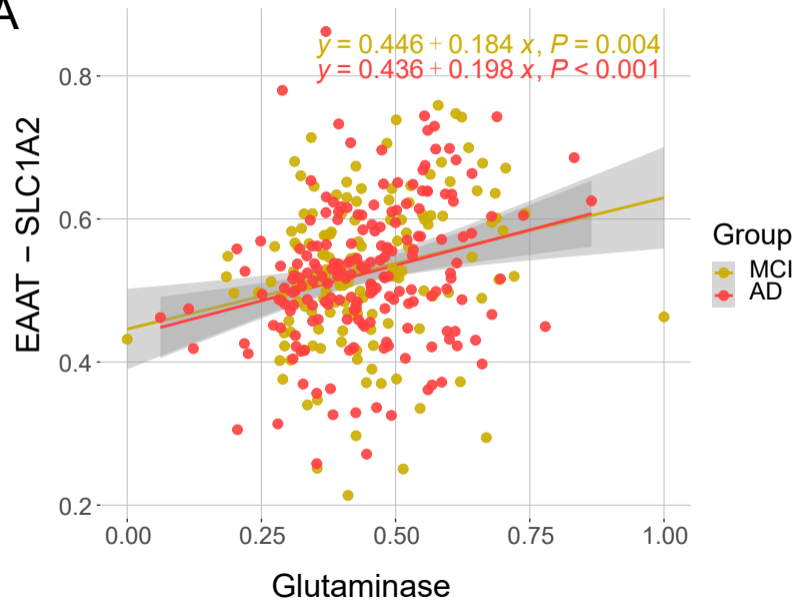

B

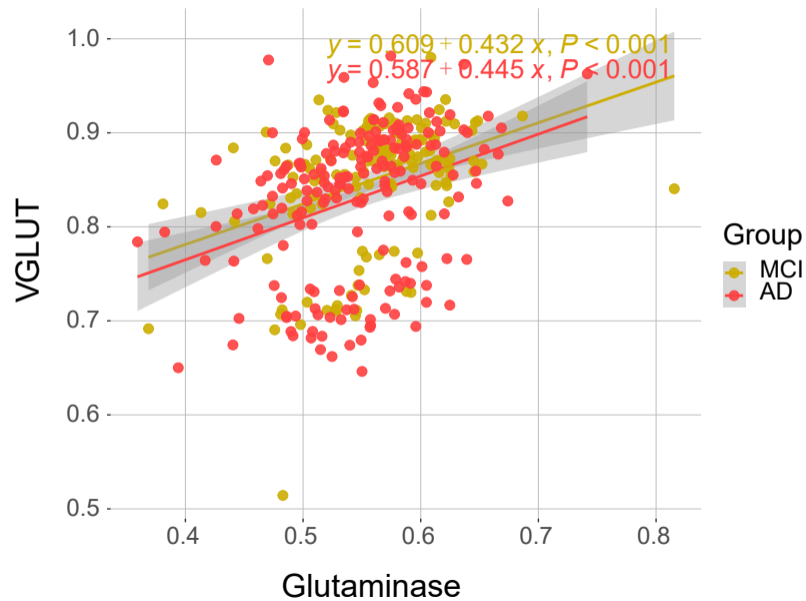

Supplement: qzaf087_Supplementary_Data [file qzaf087_supplementary_data.zip › Figure S3.pdf]

A

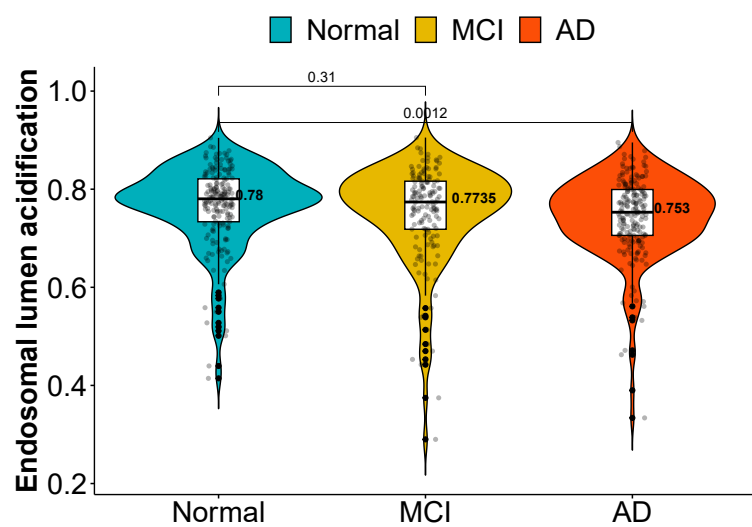

B

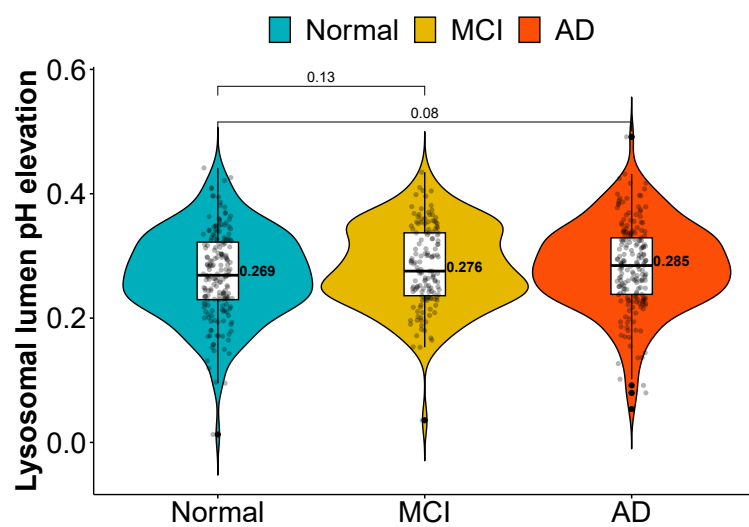

C

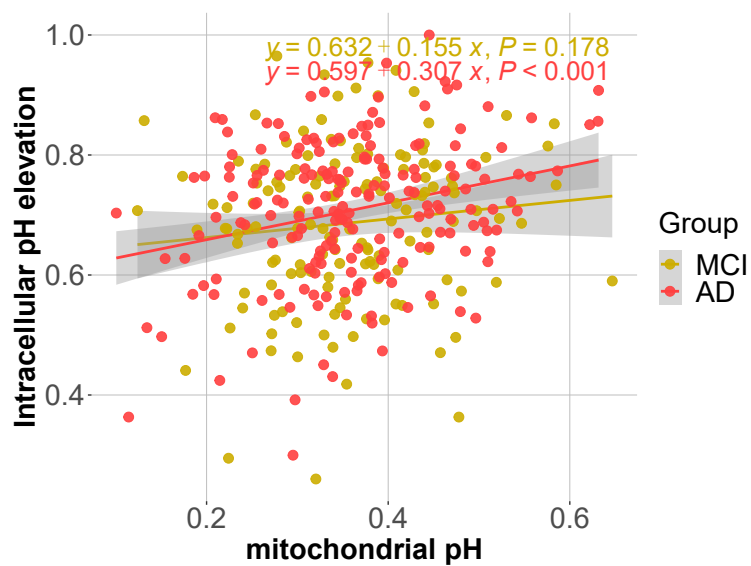

D

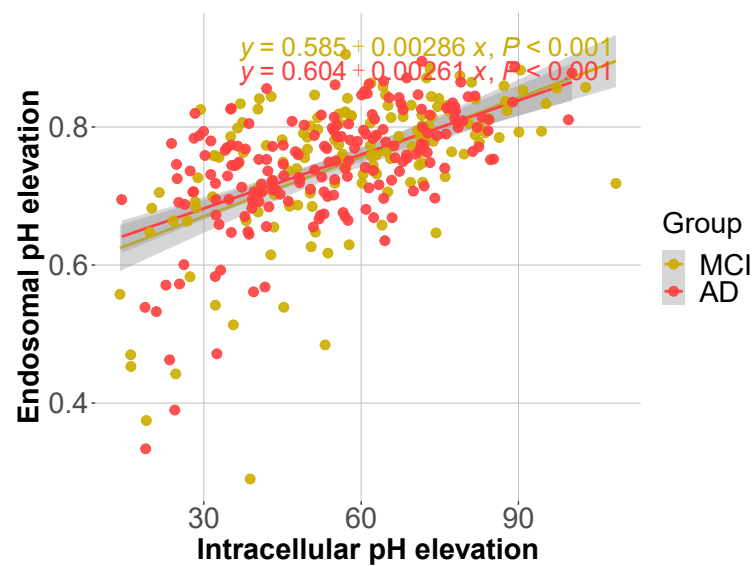

E

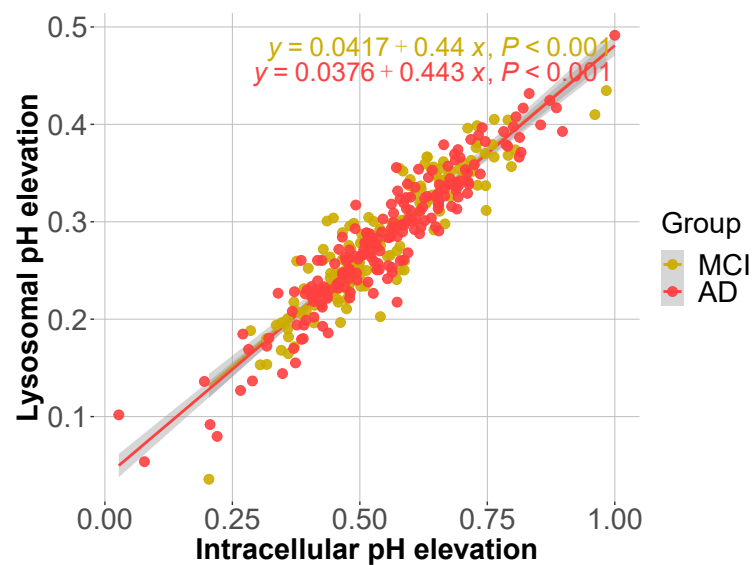

Supplement: qzaf087_Supplementary_Data [file qzaf087_supplementary_data.zip › Figure S4.pdf]

A

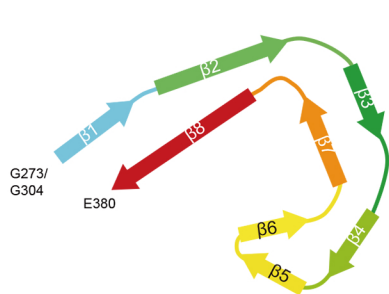

B

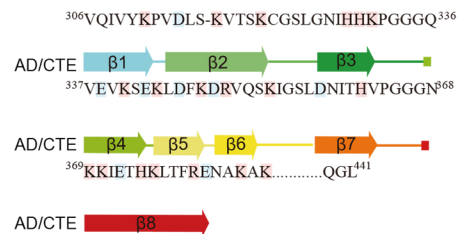

C

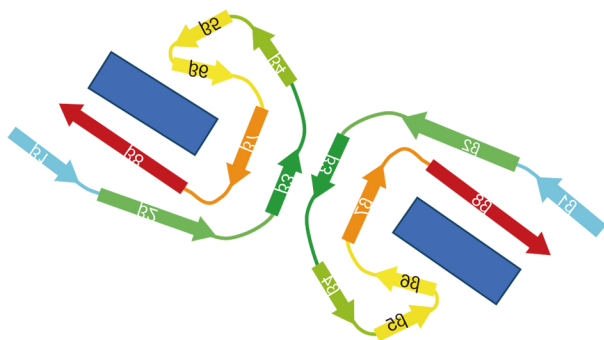

D

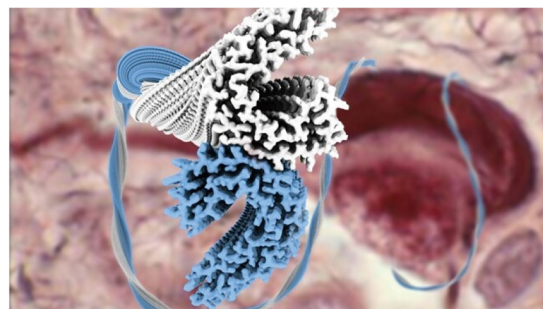

E

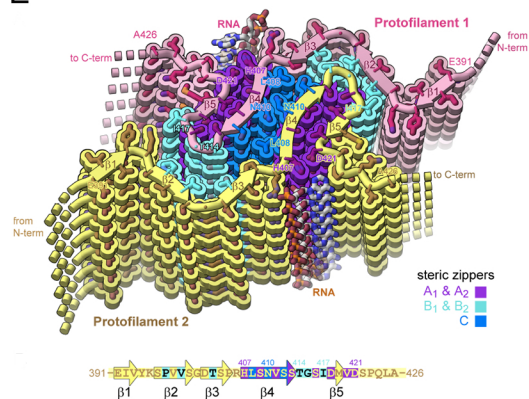

Supplement: qzaf087_Supplementary_Data [file qzaf087_supplementary_data.zip › Figure S5.pdf]

A

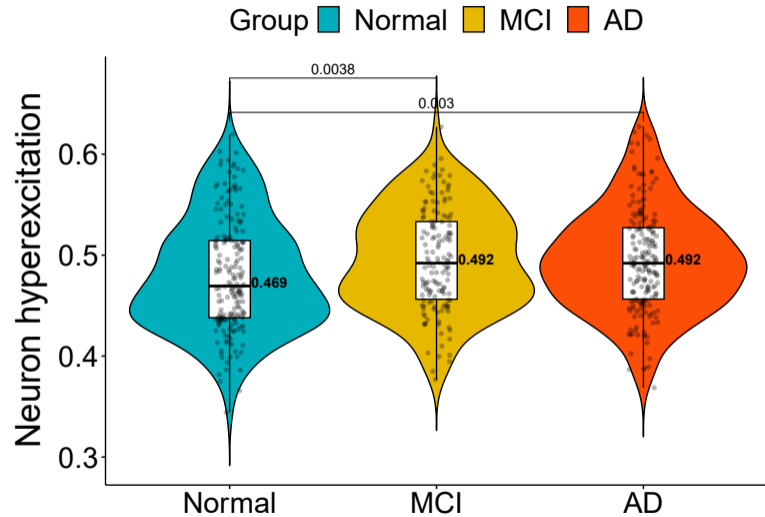

B

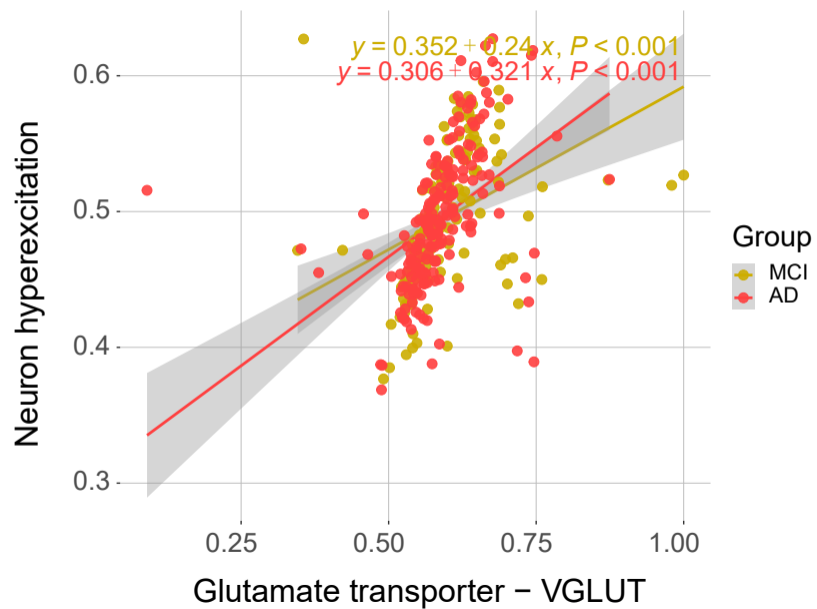

Supplement: qzaf087_Supplementary_Data [file qzaf087_supplementary_data.zip › Figure S6.pdf]

A

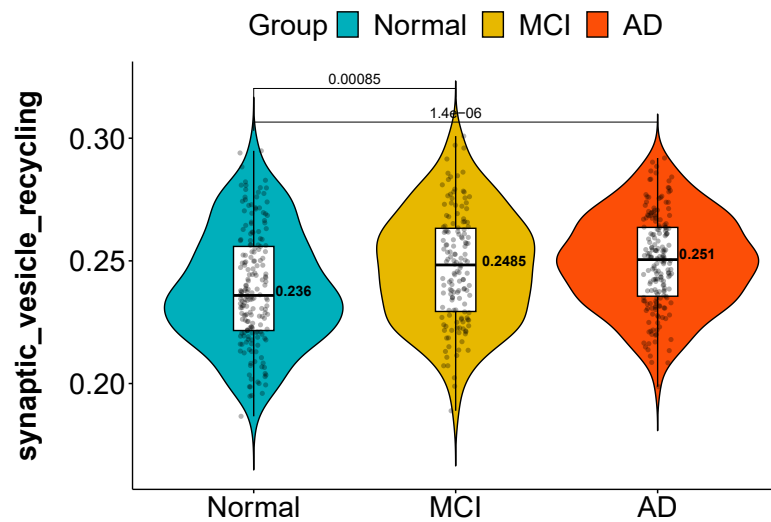

B

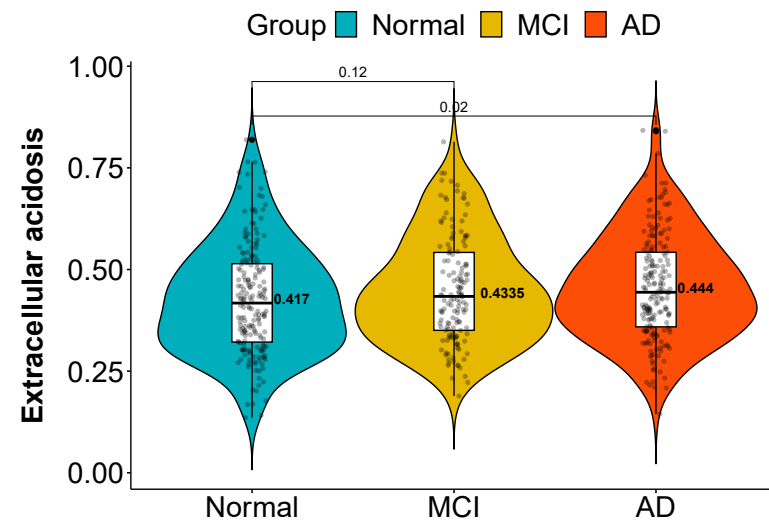

C

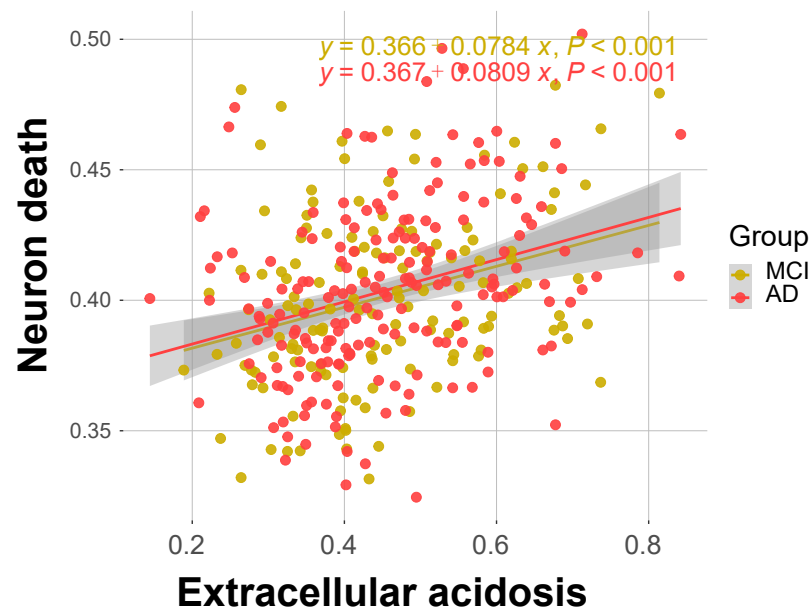

Supplement: qzaf087_Supplementary_Data [file qzaf087_supplementary_data.zip › Figure S7.pdf]

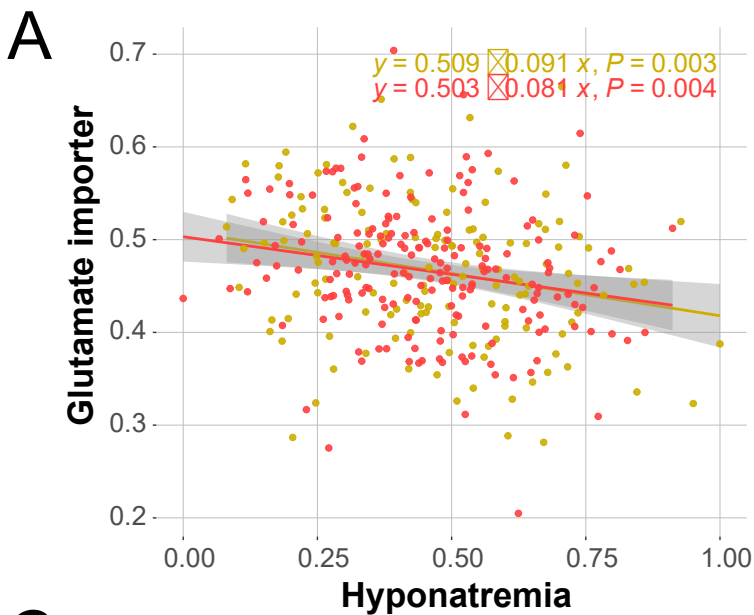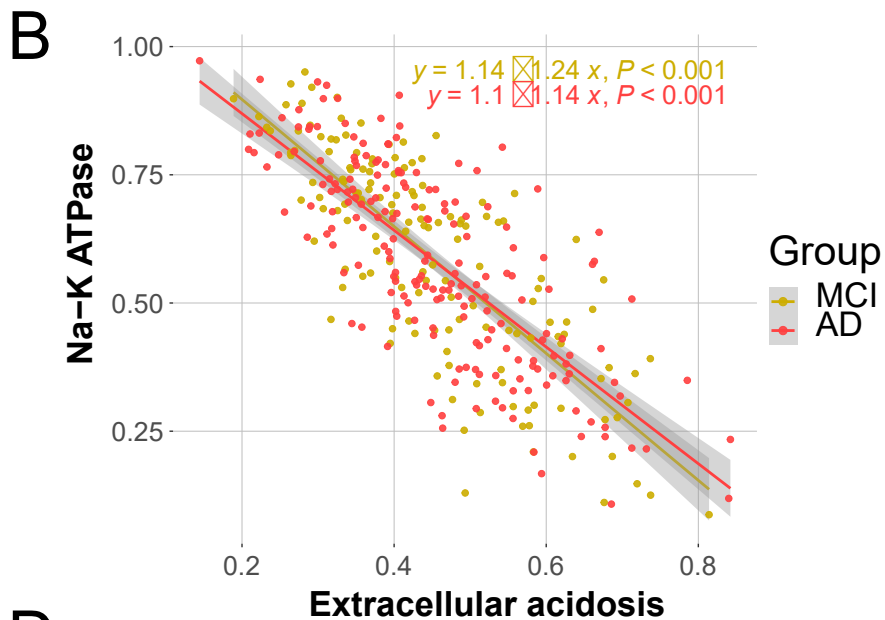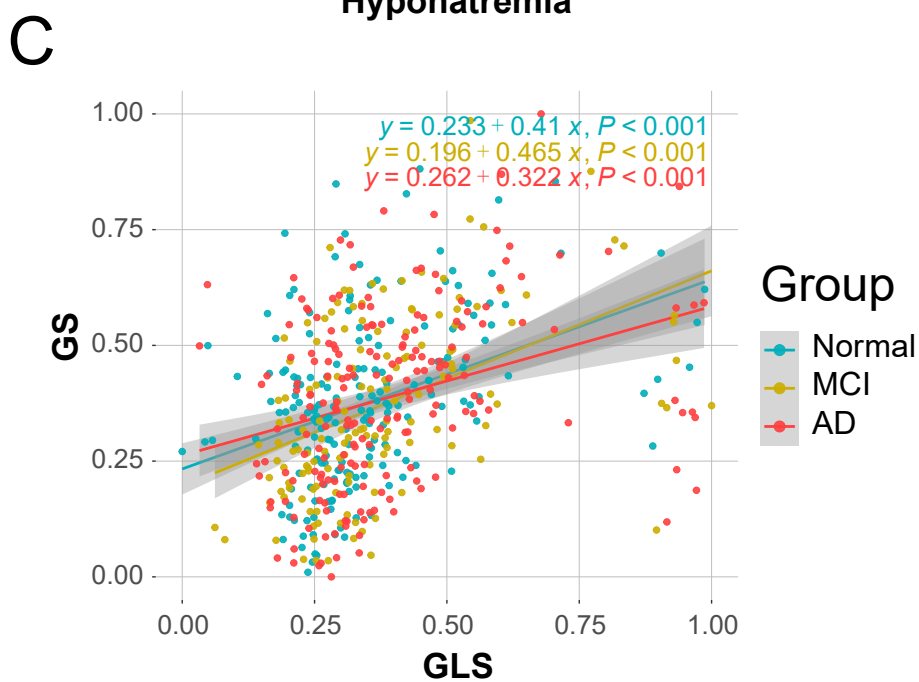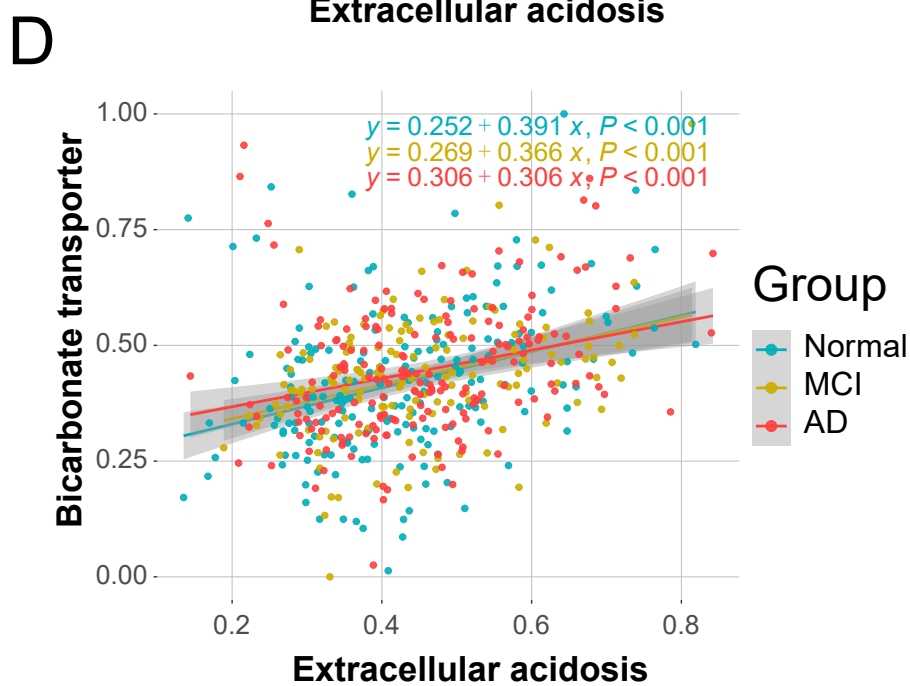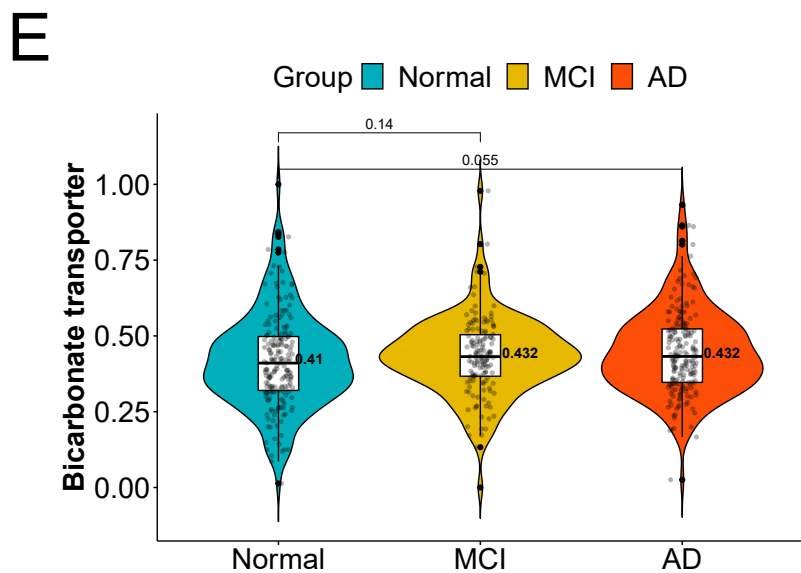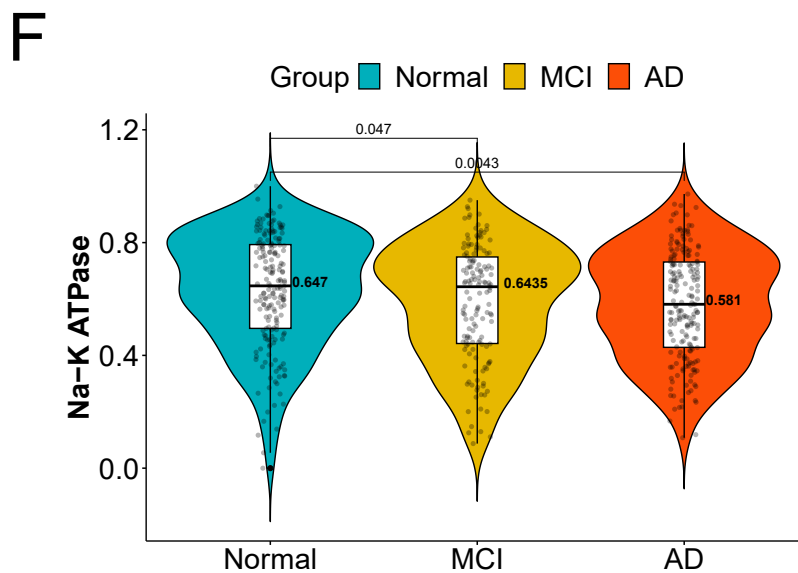

Supplement: qzaf087_Supplementary_Data [file qzaf087_supplementary_data.zip › Figure S8.pdf]

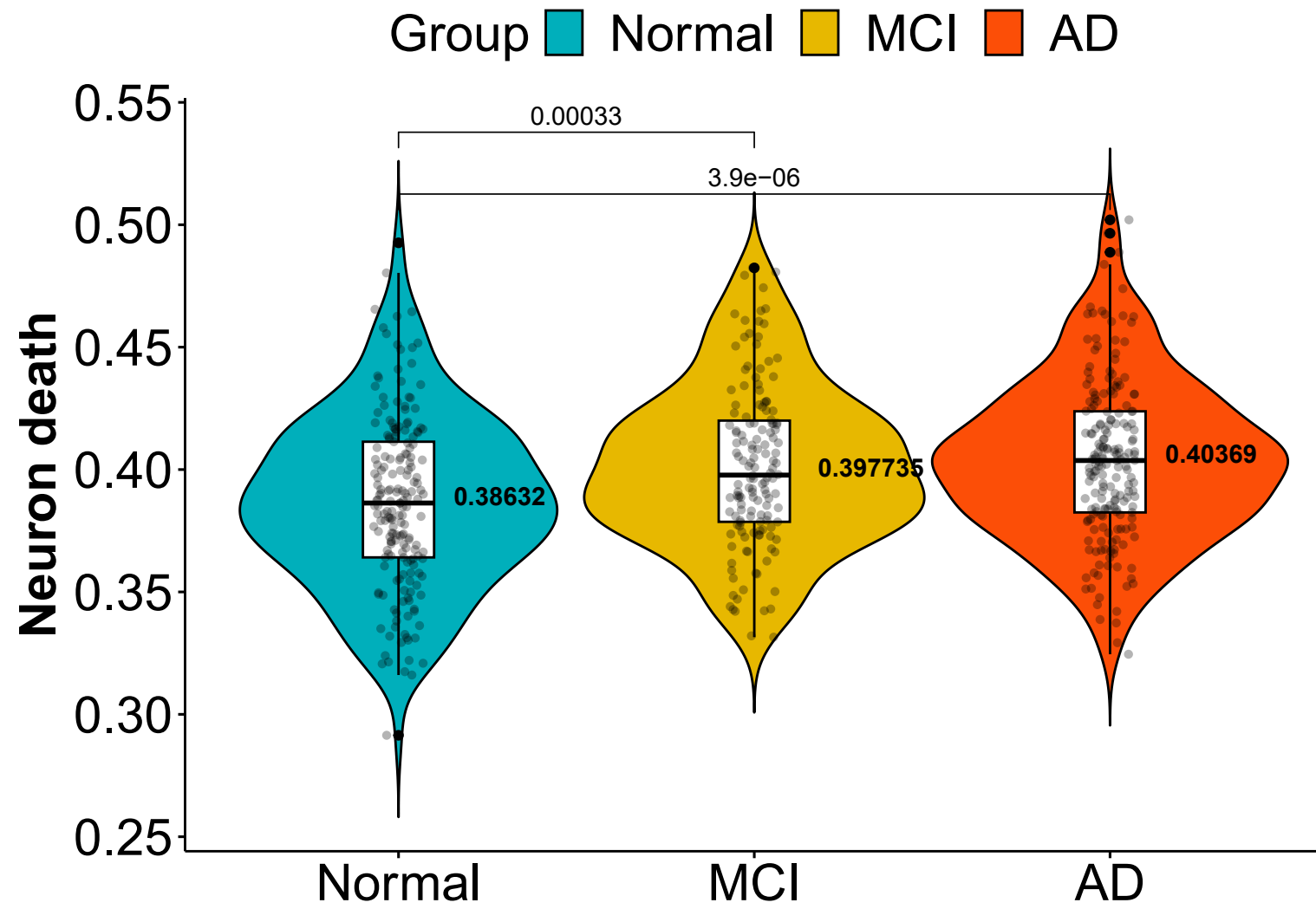

Supplement: qzaf087_Supplementary_Data [file qzaf087_supplementary_data.zip › Figure S9.pdf]
